# ω‐Hydroxyacid Dehydrogenases From *Clostridium viride*: Iron‐Containing Biocatalysts With Biotechnological Potential

**DOI:** 10.1002/cbic.70511

**Published:** 2026-08-21

**Authors:** Tarik Begic, Julia Hill, Jan L. Stitzel, Lukas Knauer, Volker Schünemann, Markus Räschle, Berta M. Martins, Antonio J. Pierik

**Affiliations:** ^1^ Biochemistry Department of Chemistry RPTU University Kaiserslautern‐Landau Kaiserslautern Germany; ^2^ Department of Physics RPTU University Kaiserslautern‐Landau Kaiserslautern Germany; ^3^ Department of Molecular Genetics RPTU University Kaiserslautern‐Landau Kaiserslautern Germany; ^4^ Institut für Biologie Humboldt‐Universität zu Berlin Berlin Germany

**Keywords:** alcohol dehydrogenase, biocatalysis, biochemistry, chemistry, Clostridium, crystal structure, electron paramagnetic resonance spectroscopy, Mössbauer spectroscopy, nonheme iron, regioselectivity

## Abstract

*ω*‐Hydroxy and oxo acids are versatile platform chemicals, accessible via enzymes of the iron‐containing alcohol dehydrogenase (FeADH) superfamily. Compared with well‐characterized zinc‐dependent ADHs, FeADHs producing such compounds remain poorly defined regarding assignment to genetic locus, biochemical and biophysical properties, and metal specificity. Here, we uncover two FeADHs from *Clostridium viride*: a highly selective 4‐hydroxybutyrate dehydrogenase and a broad‐spectrum 5‐hydroxyvalerate dehydrogenase highly active on C5–C12 substrates, identified by purification from the native organism. Chemical synthesis of 5‐oxovalerate enabled determination of kinetics for the physiological direction (*V*_max_ = 0.47 ± 0.05 kU/mg, *K*_m_ = 0.047 ± 0.011 mM). These values compare favorably with those of the oxidation (*V*_max_ = 0.13 ± 0.02 kU/mg, *K*_m_ = 0.42 ± 0.08 mM). We report the first crystal structure of an FeADH in complex with both substrate and NADH at 1.73 Å resolution. This enables identification of active‐site iron and catalytic residues, providing insight into substrate recognition. High iron occupancy in the nonreconstituted enzymes, together with Mössbauer and electron paramagnetic resonance (EPR) spectroscopic identification of high‐spin Fe^2+^, establishes iron as the native cofactor. These findings highlight the potential of *C. viride* FeADHs as attractive biocatalysts for accessing sustainable solvents, polymers, and fine chemicals via *ω*‐functionalized acids.

## Graphical abstract

*ω*‐Hydroxy and *ω*‐oxo acids are precursors to lactones, polyesters, and nylon‐6, yet selective interconverting enzymes remain scarce. *Clostridium viride* supplies two iron alcohol dehydrogenases: Cvir‐Hbd2, restricted to C4, and the genomic singleton Cvir‐Hvd, spanning C5–C12 substrates. A 1.73 Å structure resolves the His_3_Asp‐Fe^2+^ site and spectroscopy establishes high‐spin ferrous iron as the physiological cofactor.

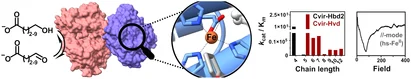

## 1 Introduction

**SCHEME 1:**
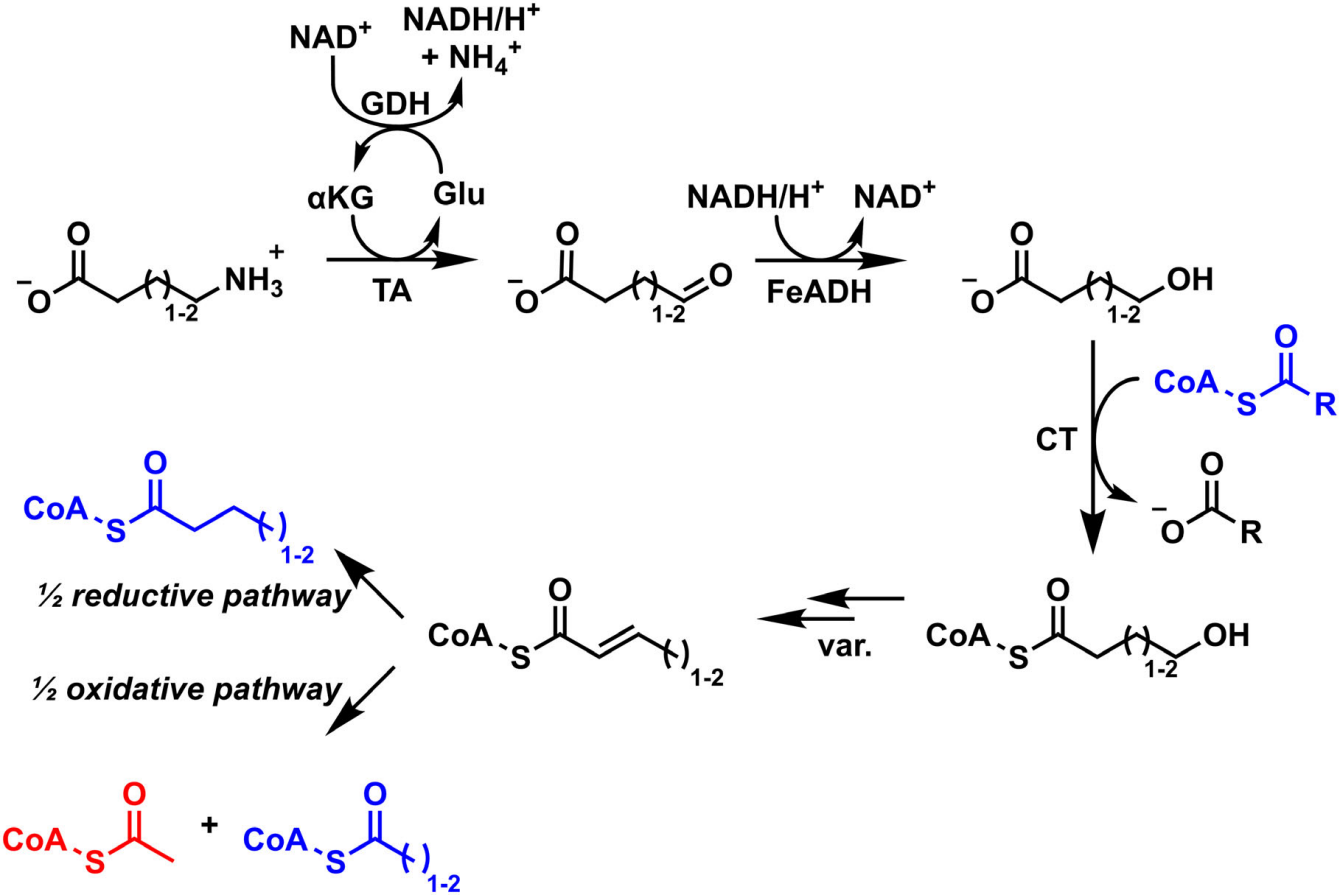
Fermentation of 4‐aminobutyrate in *C. aminobutyricum* and 5‐aminovalerate in *C. viride*. Transamination (TA) converts the *ω*‐amino acid into the corresponding *ω*‐oxo acid. Via glutamate dehydrogenase (GDH), NADH for the reduction to an *ω*‐hydroxy acid is supplied and *α*‐ketoglutarate is regenerated. After CoA transfer (CT), water elimination and Δ‐isomerization, 2‐enoyl‐CoA is formed, equally entering a reductive and an oxidative pathway, the latter yielding one ATP via substrate‐level phosphorylation of acetyl‐CoA.

Alcohol dehydrogenases (ADHs) are oxidoreductases (EC 1.1.1) that catalyze the reversible oxidation of alcohols to the corresponding aldehydes or ketones using NAD(P)^+^ as acceptor. They are classified into three groups: zinc‐dependent MDR‐ADHs (medium‐chain dehydrogenase/reductase) [1], metal‐independent short‐chain dehydrogenase/reductases [2], and FeADHs, originally named after the Fe‐dependence reported for the first characterized member, *Zymomonas mobilis* AdhB (Zmob‐AdhB) [3]. FeADHs utilize a single transition metal ion, typically coordinated by a His_3_Asp motif [4]. Structurally, FeADHs differ from MDR‐ADHs by an inverted domain organization and a predominantly *α*‐helical C‐terminal catalytic domain, unlike the mixed *α*/*β* domain of MDR‐ADHs [5]. Despite their widespread occurrence in all domains of life, only a limited number of FeADH crystal structures are available, indicating that these enzymes remain comparatively underexplored [6]. Experimental data indicate variable metal dependence among FeADHs, with some enzymes utilizing divalent cations other than iron [7, 8] or retaining activity after metal‐ion depletion [9]. These observations suggest functional flexibility and highlight the need to control metal availability and oxygen exposure during purification. FeADHs form a distinct clade within the dehydroquinate synthase (DHQ)‐FeADH superfamily, separate from fold‐related Zn^2+^‐dependent glycerol dehydrogenases [4], underscoring the need for experimental validation of function and metal ion identity. FeADHs are attractive biocatalysts as numerous thermophilic members have been characterized [10] and they catalyze a broad range of reactions, including the oxidation of *ω*‐hydroxy acids. These compounds are valuable platform chemicals for the synthesis of lactones used in green solvents, biodegradable polymers, and fine chemicals [11]. Additionally, *ω*‐oxo acids are relevant precursors for *ω*‐amino acids via transamination, allowing access to hexamethylenediamine and *ε*‐caprolactam for nylon production [12]. However, only a few FeADHs utilizing *ω*‐hydroxy‐/oxo‐acids have been characterized, including 4‐hydroxybutyrate (4HB) dehydrogenases from *Clostridium kluyveri* [13, 14] and *Cupriavidus necator* [15]. Their biochemical properties, metal dependence, and structural features remain incompletely characterized. For 5‐hydroxyvalerate (5HV), a candidate coding sequence from *Comamonas* sp. has been identified and screened for 5HV production in *Corynebacterium glutamicum* using readily available (*S*)‐lysine as the starting material [16, 17]. A key bottleneck in this route is the 5HV dehydrogenase itself, for which neither kinetic nor structural information has been reported. The MDR‐type ADH ChnD from *Acinetobacter* sp. was recently shown to oxidize medium‐chain ω‐hydroxycarboxylic acids from C6–C12 [18]. During anaerobic glutamate and proline degradation by clostridia, 4‐aminobutyrate and 5‐aminovalerate are formed, respectively. Specialized species such as *Clostridium aminobutyricum* and *Clostridium viride* (formerly *Clostridium aminovalericum* T2−7) ferment these ω‐amino acids via ω‐hydroxyacid intermediates and enoyl‐CoAs, yielding ATP through acetyl‐CoA formation (Scheme 1) [19, 20, 21]. However, genomic assignment for the enzymes of 5‐aminovalerate degradation in *C. viride* is lacking. Here we address the biochemical and structural characterization of two FeADHs from *C. viride*, including a highly selective 4HB dehydrogenase and a promiscuous 5HV dehydrogenase utilizing wide range of *ω*‐hydroxy‐carboxylic acids spanning 5–12 carbon atoms.

## 2 Results and Discussion

### 2.1 Bioinformatic Analysis of *C. viride*

**FIGURE 1:**
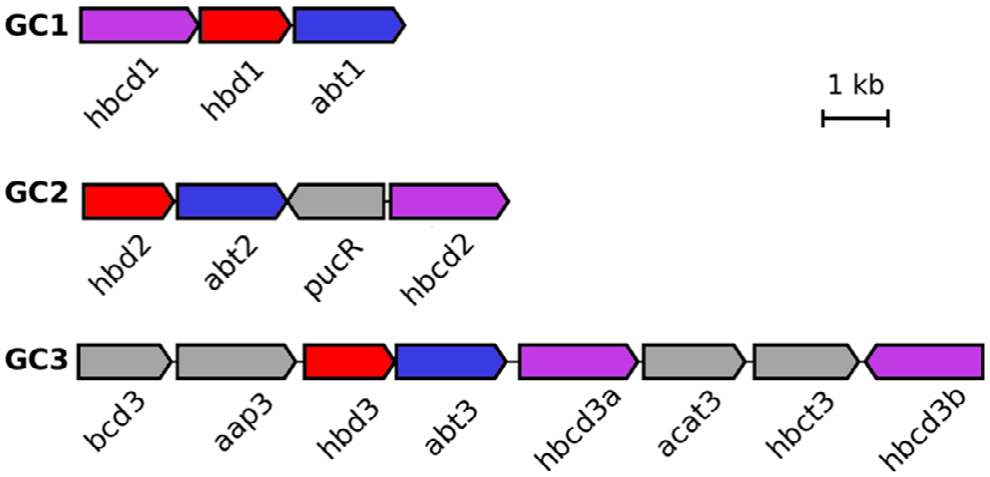
Gene clusters associated with *ω*‐amino acid fermentation in *C. viride*. Each gene cluster contains genes encoding proteins annotated as 4‐aminobutyrate transaminase (abt, in blue), 4‐hydroxybutyrate dehydrogenase (hbd, FeADH‐family, in red) and 4‐hydroxybutyryl‐CoA dehydratase (hbcd, in pink). The second cluster additionally encodes an annotated regulator (pucR), the third cluster a butyryl‐CoA dehydrogenase (bcd3), amino acid permease (aap3), acetyl‐CoA acetyltransferase (acat3), and 4‐hydroxybutyryl‐CoA transferase (hbct3).

The pipeline annotated *C. viride* genome from the “One Thousand Microbial Genomes” project was retrieved from IMG/M [22, 23]. Three gene clusters were identified, comprising annotated 4‐aminobutyrate transaminases, FeADH‐type ADHs, and 4‐hydroxybutyryl‐CoA dehydratases (Figure 1). Alignment of the three FeADHs (Cvir‐Hbd1/2/3) with characterized FeADHs, including *Zymomonas mobilis* AdhB (Zmob‐AdhB) and *C. necator* 4HB dehydrogenase (Cnec‐Hbd) showed conservation of the metal‐coordinating His_3_Asp motif (Figure S1). Cvir‐Hbd1/2/3 share 68%–74% sequence identity, compared with ~50% and ~23%, respectively, to the characterized *C. kluyveri* and *C. necator* 4HB dehydrogenases, respectively. Given the established 5‐aminovalerate metabolism in *C. viride*, we hypothesized that at least one of these gene clusters is responsible for the 5‐aminovalerate fermentation.

### 2.2 Expression and Purification of 4HB Dehydrogenases

Cvir‐Hbd1/2/3 were recombinantly expressed in *E. coli*, His_6_‐purified, Mn^2+^‐reconstituted to minimize oxygen sensitivity, and screened for activity toward 4HB and 5HV (Figure S2). All three enzymes showed very low activity toward 5HV (*k*_cat_/*K*_m_ = 1−2 s^−^^1^ M^−1^), but values for 4HB oxidation (*k*_cat_/*K*_m_ = 3−9 * 10^3^ s^−^^1^ M^−1^) were comparable with authentic 4HB dehydrogenases [14, 15]. Thus, Cvir‐Hbd1/2/3 are genuine 4HB dehydrogenases, likely linked to 4‐aminobutyrate fermentation, and Cvir‐Hbd2 was selected for detailed kinetic and structural analysis due to its highest specific activity. To avoid potential incorporation of nickel from Ni‐NTA into the active site, and to obtain native protein for both kinetic measurements and structure determination, we chose an intein‐based affinity system. The enzyme is expressed with the N‐terminal ProteinSelect tag, which binds to the Cytiva ProteinSelect‐material and is autocatalytically cleaved [24]. Tag‐free protein of the expected mass was recovered after 2–4 h at 23 °C (Figure S3). Purified Cvir‐Hbd2 contained 0.68 ± 0.11 Fe ions and 0.75 ± 0.05 nicotinamide dinucleotides, mainly NADH, per monomer (Table 1). Unlike aerobically Ni‐NTA‐purified enzyme, anaerobically isolated Cvir‐Hbd2 reached specific activities of up to 100 U/mg with sodium 4HB.

**TABLE 1:** Biochemical and kinetic parameters of Cvir‐Hbd2, Cvir‐Hvd, and variants. As Cvir‐Hvd‐D39G was not soluble, no data is shown. All values represent mean ± SEM from two independent protein preparations, except for D194A/H198A. *V*_max_ (U/mg) is converted to *k*_cat_ (s^−1^) by multiplication with a factor of 0.683, corresponding to the molecular mass of the protein (41 kDa) divided by 60.

Variant	Fe/monomer	NAD^+^/monomer	NADH/monomer	Substrate	*V*_max_, U/mg	*K*_m_, mM	*k*_cat_/*K*_m_, s^−^^1^ M^−1^
Hbd2							
WT	0.68 ± 0.11	0.25 ± 0.01	0.51 ± 0.05	4HB	117 ± 21	0.80 ± 0.15	1.0 * 10^5^
				5HV	45 ± 4	50 ± 10	6.1 * 10^2^
Y256A	0.38 ± 0.08	0.06 ± 0.06	0.58 ± 0.08	4HB	44 ± 6	~14	2.1 * 10^3^
N270A	0.86 ± 0.04	0.15 ± 0.01	0.37 ± 0.01	4HB	22 ± 3	0.28 ± 0.05	5.4 * 10^4^
R348A	0.54 ± 0.02	0.03 ± 0.02	0.41 ± 0.04	4HB	126 ± 24	41 ± 5	2.1 * 10^3^
R348E	0.46 ± 0.01	0.37 ± 0.10	0.41 ± 0.01	4HB	>50	>50	<7 * 10^2^
Hvd							
WT	0.54 ± 0.01	0.38 ± 0.38	0.41 ± 0.08	5HV	125 ± 19	0.42 ± 0.08	2.0 * 10^5^
				5OV	468 ± 52	0.047 ± 0.011	6.8 * 10^6^
D194A/H198A	0.01	0.21	0.63	5HV	<0.01	ND	ND

Abbreviations: ND, not determined; WT, wildtype.

### 2.3 Crystallographic Analysis

**FIGURE 2:**
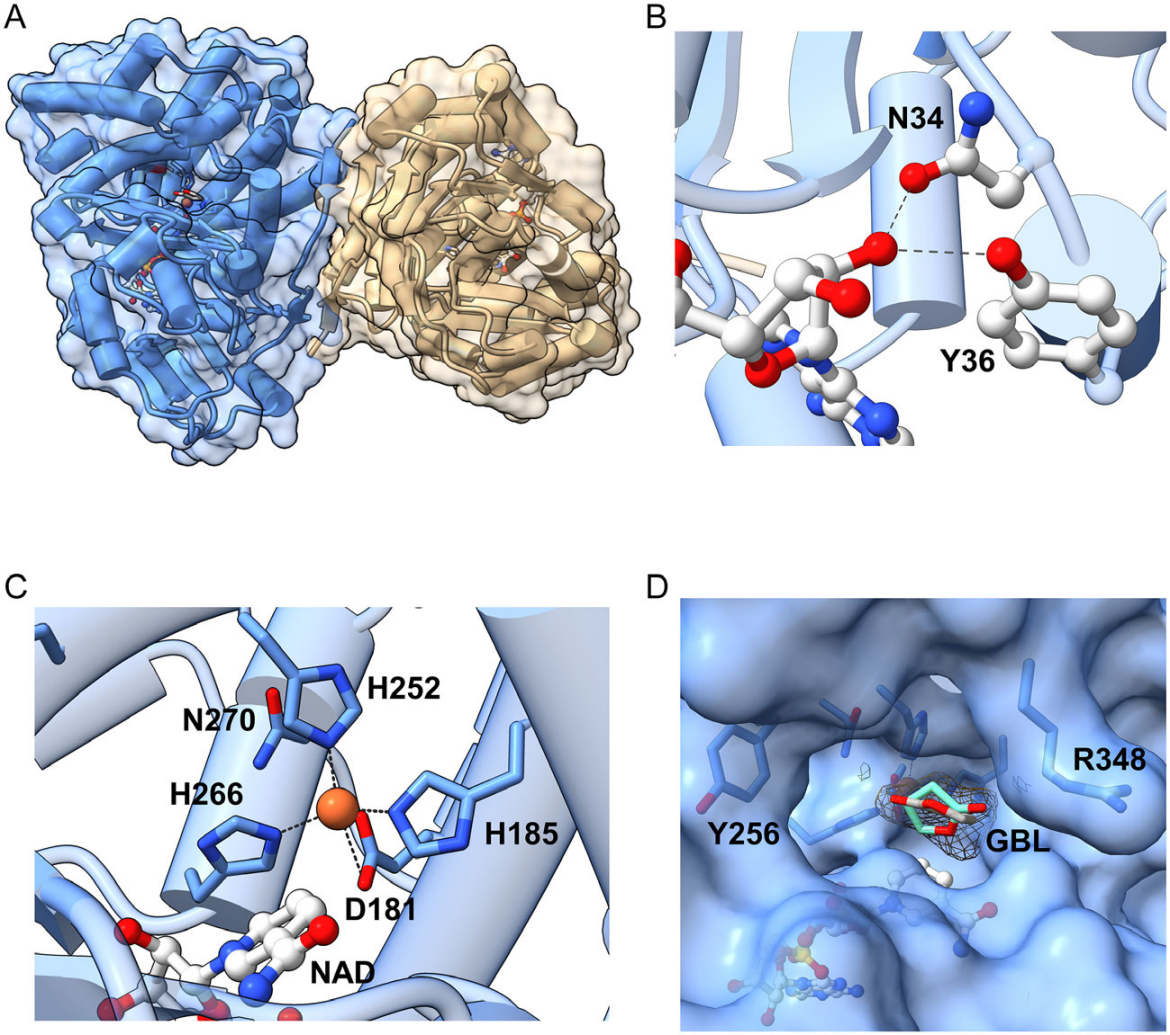
Structural analysis of 4‐hydroxybutyrate dehydrogenase (Cvir‐Hbd2), PDB 30SM. (A) The enzyme forms a homodimer stabilized by an N‐terminal *β*‐sheet interface, assembled by two N‐terminal *β‐*strands from each monomer. (B) NAD(H)‐binding pocket highlighting residues N34 and Y36 at H‐bonding distance (dashed lines). These interactions may contribute to cofactor specificity, as NADP^+^ carries an additional 2′‐phosphate group at this position. (C) The active site contains an iron‐center coordinated by D181, H185, H252, and H266 in proximity to the NAD(H) cofactor. Additionally, the noncoordinating N270 is located within 3.7 Å of the iron ion. (D) Electron density corresponding to two alternative conformations of *γ*‐butyrolactone (GBL) is present in the substrate tunnel leading to the active site. The *F*_o_–*F*_c_ difference electron density map, prior to ligand modeling, is shown as a mesh (contour level: 1.5 rmsd). The putative catalytic base Y256 is oriented away from the active site, whereas the flexible R348 is positioned at the beginning of the tunnel and may participate in substrate binding.

For structural analysis, Cvir‐Hbd2 was reconstituted with Fe^2+^ and subjected to gel filtration. Data from crystals formed in the presence of NADH and sodium 4HB by sitting‐drop vapor diffusion were refined to a resolution of 1.73 Å (Figure 2A). The asymmetric unit cell contains two independent polypeptide chains that build a homodimer. Omit maps indicated the presence of the cocrystallized NADH cofactor and a metal ion in each of the two polypeptide chains. The average B value for the main chain, side chains, and NADH cofactor of the A monomer (22.6, 26.1, and 21.2 Å^2^, respectively) was lower compared to the B monomer (25.3, 29.0, and 23.7 Å^2^) (Table S3). We therefore focus on the A monomer in the following structural description. A zipper‐like arrangement of two N‐terminal *β*‐sheets from each monomer forms the dimer interface, burying an area of 1044 Å^2^ corresponding to ~7% of each monomer's molecular surface. Thus, the crystal structure shows the physiological quaternary structure in agreement with gel filtration (Figure S4). Cvir‐Hbd2 contains the conserved FeADH fold, with an N‐terminal Rossmann domain and a C‐terminal *α*‐helical domain incorporating the metal‐binding site. FeADH cofactor specificity depends on a conserved residue in the Rossmann fold (position 39 in Zmob‐ADH2). Aspartate favors NAD^+^, whereas glycine and threonine are associated with NADP^+^ and dual specificity, respectively [4]. *C. viride* and other clostridial 4HB dehydrogenases (cd14860 subfamily [25]) contain an asparagine at this position (N34 in Cvir‐Hbd2; Figures 2B and S5). The crystal structure shows N34 and Y36 forming H bonds (<3 Å) with the 2′‐OH group of the adenosine ribose of NAD(H). The iron ion is coordinated by H185, H252, H266 (all N_ε_), and D181 (Figure 2C), and its identity defined by the anomalous signal at the Fe absorption K‐edge (Figure S6A). In proximity (<4 Å), NAD(H) and a conserved asparagine residue (N270) are located, the latter being present in multiple members of this enzyme family but, to our knowledge, not previously discussed in detail. A tunnel leading to the active site is observed in the Cvir‐Hbd2 structure, with electron density at the end of the tunnel consistent with the lactone form of 4HB (GBL) (Figure 2D). In several FeADHs, a conserved histidine is located near the active site but oriented away from the metal center, which has been proposed to act as a catalytic base [5, 15]. In Cvir‐Hbd2, this position is occupied by Y256, which is also positioned close to the substrate‐access tunnel but points away from the bound substrate. At the tunnel entrance, the guanidinium group of R348 may contribute to the recruitment of the *ω*‐carboxylate moiety of the substrate. The poorly defined electron density of R348 shows that it is highly flexible. One of its rotamers could potentially bind the nonlactonized 4HB (Figures S6B and S7).

### 2.4 Kinetic and Biochemical Parameters of 4HB Dehydrogenase and Its Variants

**FIGURE 3:**
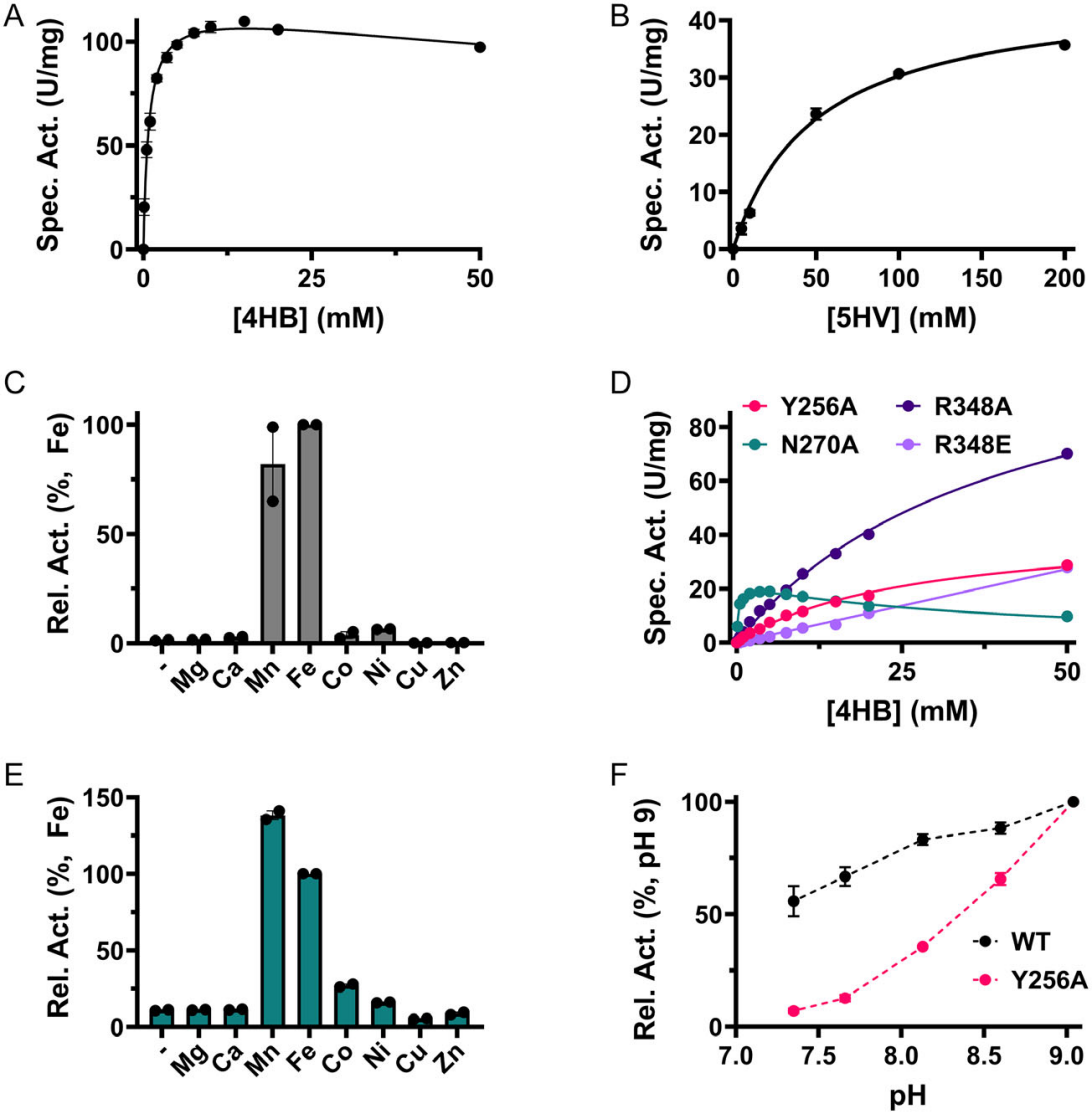
Activity profiling of Cvir‐Hbd2 and selected active‐site variants. (A) Michaelis–Menten analysis with 4HB at constant NAD^+^ concentration (1 mM; 100 mM Tris/HCl, pH 9.0). (B) Michaelis–Menten analysis with 5HV under the same conditions as in A. (C) Activity of the wildtype apoenzyme after reconstitution with different MCl_2_ salts, measured with 5 mM 4HB and 1 mM NAD^+^ in 100 mM Tris/HCl, pH 9.0 Activities are shown relative to Fe^2+^‐reconstituted enzyme (179 ± 5 U/mg). (D) Michaelis–Menten analysis of selected active site mutants for 4HB‐oxidation under the same conditions as in A. (E) Activity of the N270A‐apoenzyme after reconstitution with different MCl_2_ salts under the same conditions as in C. Activities are shown relative to Fe^2+^‐reconstituted enzyme (8.6 ± 0.1 U/mg). (F) pH‐dependence of 4HB‐oxidation activity for the wildtype and Y256A (20 mM 4HB, 1 mM NAD^+^, 100 mM Tris/HCl, pH 9.0). Kinetic parameters are summarized in Table 1.

Untagged, iron‐reconstituted Cvir‐Hbd2 displayed a *V*_max_ of 117 ± 6 U/mg with a *K*_m_ of 0.80 ± 0.15 mM for the oxidation of 4HB with NAD^+^ (*k*_cat_/*K*_m_ = 10^5^ s^−^^1^ M^−1^). At higher 4HB concentrations, a slight inhibition was observed (*K*_i_ > 0.2 M) (Figure 3A). Using NADP^+^, we observed three orders of magnitude lower specific activity. For 5HV, catalytic efficiency was below 1% of that observed for 4HB oxidation (*k*_cat_/*K*_m_ ~600 s^−^^1^ M^−1^) (Figure 3B). EDTA led to a concentration‐dependent inactivation with less than 5% activity remaining at 10 mM (Figure S8A). Iron removal proceeded more efficiently (<2% residual activity) at lower protein concentration after several hours (Figure S8B). After removal of excess EDTA by gel filtration, the apo‐enzyme was reconstituted in a defined manner with various divalent metal ions (Figure 3C). In contrast to the slow iron ion removal (>5 h), very rapid iron ion incorporation was observed (<1 min), based on activity measurements. Similar activities were obtained upon Fe^2+^‐ and Mn^2+^‐reconstitution, whereas Co^2+^‐ and Ni^2+^‐reconstitution resulted in minor activity gain (3%–6%). Addition of Cu^2+^ or Zn^2+^ further decreased the residual activity, in accordance with reports of strong zinc binding in the active site of FeADHs [26].

Based on the crystal structure, four variants (Y256A, N270A, and R348A/E) were analyzed with respect to activity and substrate affinity for 4HB oxidation (Figure 3D). All were expressed and purified to homogeneity (Figure S9). The N270A‐variant was purified with near‐stoichiometric iron content (0.86 ± 0.04 Fe/monomer; Table 1), but retained only ~20% of wild type activity. It showed increased substrate affinity and stronger substrate inhibition (*K*_m_ = 0.28 ± 0.05 mM; *K*_i_ = 37 ± 6 mM). Though the specific activity was much lower, the relative activity of manganese‐ versus iron‐reconstituted enzyme after iron‐removal was higher (Figure 3E). This points to an indirect role of this residue in tuning the metal site for catalysis. For the Y256A variant we observed reduced turnover (*V*_max_ = 44 ± 6 U/mg) and substrate affinity (*K*_m_ ~ 14 mM), accompanied by a very pronounced loss of activity at lower pH compared to the wild type (Figure 3F). The ~50‐fold decrease of *k*_cat_/*K*_m_ indicates that Y256 is directly or indirectly involved in deprotonation of the substrate alcohol, resulting in decreased formation of the alkoxide and thus weaker substrate binding. Modification of the tunnel residue R348 supported its role to recruit 4HB. While R348A retained comparable activity to the wild type, substrate affinity strongly decreased (*K*_m_ = 41 ± 5 mM). The charge‐reversal variant R348E exhibited an even lower apparent affinity, with no saturation reached within the experimentally tested substrate range (*K*_m_ > 50 mM). These findings are consistent with a role of R348 in binding the carboxylate moiety of the substrate.

### 2.5 Purification of 5HV Dehydrogenase From Its Original Source

Earlier work reported a dedicated NAD^+^‐dependent 5HV dehydrogenase in *C. viride* with kinetic parameters distinct from Cvir‐Hbd1/2/3 [19]. As bioinformatic search did not indicate an obvious candidate, we decided to purify the 5HV activity from 5‐aminovalerate grown *C. viride*. Via anion‐exchange chromatography followed by hydrophobic interaction chromatography, the fractions containing 5HV dehydrogenase activity had a prominant 41 kDa protein band (Figures S10 and S11). Mass spectrometry identified an FeADH family enzyme as the second most abundant hit after glutamate dehydrogenase. The corresponding gene is not part of any identifiable gene cluster, indicating that no dedicated genomic organization for 5‐aminovalerate metabolism is present in *C. viride*. Bioinformatic analysis revealed the NAD^+^‐associated Rossmann‐fold aspartate and conserved metal‐coordinating residues. Unlike Cvir‐Hbd2, Cvir‐Hvd contains a histidine at the Y256‐equivalent position, as in most characterized FeADHs [4]. Cvir‐Hvd shares only ~31% sequence identity with the proposed *Comamonas* sp. 5HV dehydrogenase CpnD.

### 2.6 Biochemical Parameters of 5HV Dehydrogenase

Unfortunately, recombinant expression in *E. coli* and purification of Twin‐Strep‐tagged Cvir‐Hvd with the Strep‐Tactin XT system yielded heterogeneous protein with three bands, possibly due to partial proteolysis of the tag within the oligomer (Figure S12). Therefore, Cvir‐Hvd was purified using the Cytiva ProteinSelect system, already successfully used for Cvir‐Hbd2. However, whereas Cvir‐Hbd2 was recovered within 2 h after incubation at 23 °C, Cvir‐Hvd required overnight incubation for efficient tag cleavage and elution (Figure S13). Size‐exclusion chromatography indicated an apparent molecular weight of >200 kDa, suggesting a hexameric or higher‐order oligomer. This quarternary structure with multiple subunits requires complete intein cleavage for release, explaining the longer intein cleavage.

Similar to Cvir‐Hbd2, Cvir‐Hvd copurified with iron (0.54 ± 0.01 Fe/monomer) and NAD(H) (0.8 ± 0.3 per monomer). Circular dichroism (CD) spectroscopy indicates binding of NAD(H) in the active site by an pronounced extrinsic Cotton effect (Figure S15). Addition of NADH enhanced the CD signal, while addition of 5‐oxovalerate (5OV) led to loss of the 260 and 340 nm signal, consistent with dissociation of the formed NAD^+^. Modification of the putative NAD^+^‐specificity D39‐residue to glycin abolished solubility, suggesting that NAD^+^‐cofactor binding is critical for folding (Figure S14). Abolition of the conserved DXXXH‐metal‐binding motif (D194A/H198A) yielded soluble protein that was essentially iron‐free but still copurified with near‐stoichiometric NAD(H).

### 2.7 Kinetic Parameters of 5HV Dehydrogenase

**FIGURE 4:**
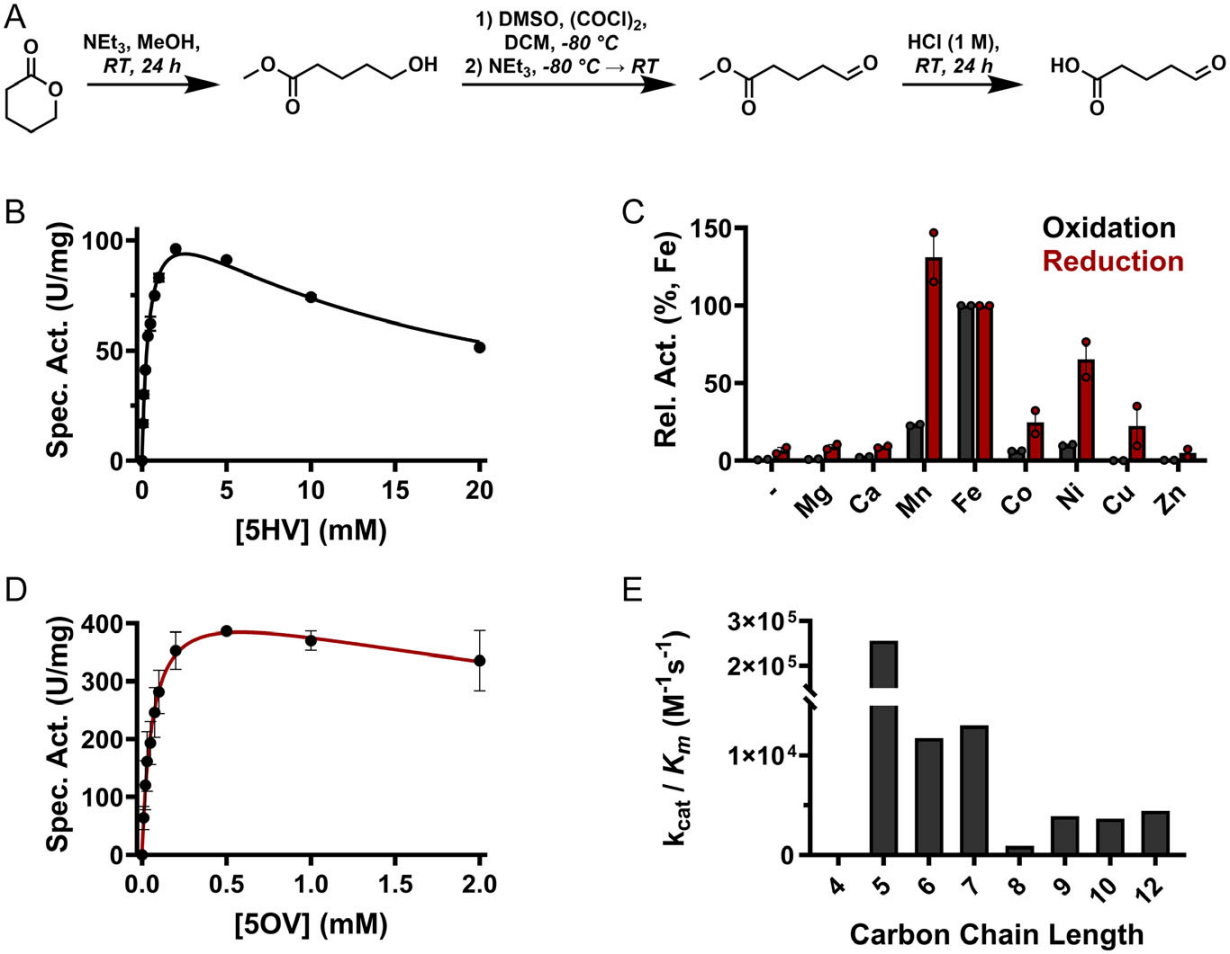
Synthesis of 5‐oxovalerate and activity profiling of Cvir‐Hvd. (A) Synthesis of 5‐oxovalerate from *δ*‐valerolactone by base‐catalyzed ring opening in methanol, Swern oxidation, and acid‐catalyzed ester hydrolysis. Detailed procedures are provided in the Supporting Information. (B) Michaelis–Menten analysis with iron‐reconstituted enzyme for 5 HV‐oxidation at constant NAD^+^ concentration (1 mM; 100 mM Tris/HCl, pH 9.0). (C) Activity after reconstitution with different MCl_2_ salts for alcohol oxidation (5 mM 5 HV and 1 mM NAD^+^ in 100 mM Tris/HCl pH 9.0) or aldehyde reduction (2 mM 5OV and 0.1 mM NADH in 100 mM BisTris/HCl pH 6.0). Activities are shown relative to Fe^2+^. (D) Michaelis–Menten analysis with iron‐reconstituted enzyme for 5OV‐reduction at constant NADH concentration (0.1 mM; 100 mM BisTris/HCl, pH 6.0). (E) Catalytic efficiencies with *ω*‐hydroxy acids of different chain lengths. Kinetic parameters are summarized in Tables 1 and S1.

In the 5‐aminovalerate fermentation of *C. viride*, the 5OV formed by transaminase action is reduced to the corresponding alcohol. To characterize the physiological reaction, the noncommercially available aldehyde substrate was chemically synthesized via base‐catalyzed ring opening of *δ*‐valerolactone, followed by Swern oxidation and subsequent ester hydrolysis (Figure 4A). Activation of Cvir‐Hvd by various metal ions was assessed for both the oxidative and reductive reactions. Alcohol oxidation was only significant for Fe^2+^ and Mn^2+^, as observed for Cvir‐Hbd2, but the relative activity for manganese was lower (Figure 4B). For the physiological direction of aldehyde reduction, specific activities with iron were fourfold higher than for the oxidative direction, with Mn^2+^ slightly outperforming Fe^2+^ (1.3‐fold). Additionally, Ni^2+^‐, Cu^2+^‐, and Co^2+^‐reconstitution yielded substantial activity only for the reductive direction, while Zn^2+^ remained inhibitory. Alanine replacement of the DXXXH‐metal‐binding motif abolishes enzymatic activity, in accordance with other enzymes of this family [15].

Kinetic analysis of the Fe^2+^‐reconstituted Cvir‐Hvd revealed a *V*_max_ of 125 ± 19 U/mg and *K*_m_ of 0.42 ± 0.08 mM for sodium 5HV, including substrate inhibition (*K*_i_ = 15 ± 4 mM) (Figure 4C). With the manganese‐reconstituted enzyme, 10‐fold decreased specific activity for 5 HV‐oxidation with comparable affinity (*V*_max_ = 9.5 ± 1 U/mg, *K*_m_ = 0.69 ± 0.13 mM, *K*_i_ = 14 ± 3 mM) was measured under aerobic conditions (Figure S16A). Maximum activity was observed at NAD^+^‐concentrations >1 mM (*K*_m_ = 0.14 ± 0.02 mM) with substrate binding occuring via an ordered Bi–Bi mechanism (Figure S16B,C). For the reduction of 5OV, the enzyme exhibited a very high *k*_cat_/*K*_m_ of 6.3 * 10^6^ s^−^^1^ M^−1^ (Figure 4D), consistent with its physiological function (*V*_max_ = 468 ± 52 U/mg, *K*_m_ = 47 ± 11 µM, *K*_i_ ~ 5 mM). Substrate profiling for alcohol oxidation showed that the specificity constant toward 4HB is very low (0.02% of *k*_cat_/*K*_m_ for 5 HV), while the longer‐chain substrates (C6–C7, C9–C12) were accepted as substrates (~2%–5%) (Figure 4E; values in Table S1). Notably, 8‐hydroxyoctanoate obtained from different suppliers showed low activity (~0.4%). The specificity constants are comparable to those reported for *Acinetobacter* sp. ChnD, with Cvir‐Hvd displaying approximately one order of magnitude higher specific activities, particularly with 6‐hydroxycaproate [18].

### 2.8 Stereospecificity of NADH Hydride Transfer

**FIGURE 5:**
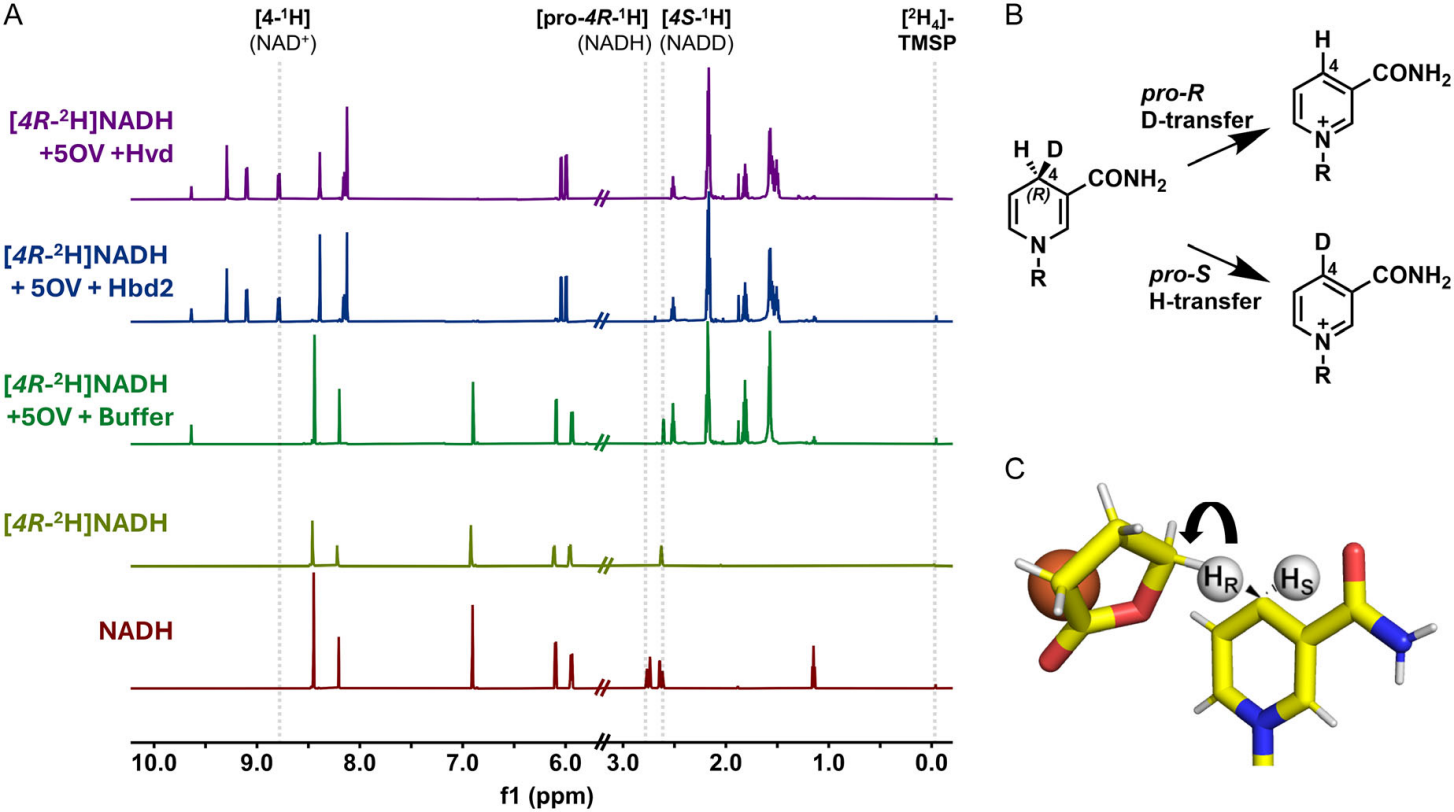
Stereospecificity of hydride transfer for Cvir‐Hbd2 and Cvir‐Hvd. (A) ^1^H‐NMR (600 MHz, water suppression) analysis of NADH, [4*R*‐^2^H]‐NADH (NADD), [4*R*‐^2^H]‐NADH with 5OV in buffer as control, and upon addition of either Cvir‐Hbd2 or Cvir‐Hvd. (B) Expected products after reaction of [4*R*‐^2^H]‐NADH with 5OV: pro‐*R* transfer yields [C4‐^1^H]‐NAD^+^, pro‐*S* transfer yields [C4‐^2^H]‐NAD^+^. (C) Position of NADH relative to *γ*‐butyrolactone in the crystal structure of Cvir‐Hbd2, suggesting transfer of the pro‐*R* hydride of NADH to the substrate (PDB 30SM).

Within the FeADH family, stereospecificity of hydride‐transfer was experimentally determined for Zmob‐Adh2 and Ecol‐FucO [27, 28], but pro‐*R* transfer has been only structurally proven for Ecol‐FucO [29]. To broaden the mechanistic knowledge and to corroborate our crystallographic analysis, we used ^1^H‐NMR to assess stereospecificity of hydride transfer in both characterized FeADHs of *C. viride*. 5OV was used as a readily accessible hydride acceptor for both enzymes. Stereospecifically deuterated NADH ([4*R*‐^2^H]NADH, NADD) was produced by reduction of NAD^+^ using deuterated ethanol catalyzed by the pro‐*R‐*specific yeast ADH1 [30]. Upon hydride transfer to an excess of 5OV, the signal at C4 of NADD (singlet at 2.6 ppm) disappeared, indicating completion of the reaction (Figure 5A). For both tested FeADHs, the proton signal at C4 of NAD^+^ (doublet at 8.8 ppm) remained, indicating pro‐*R* hydride transfer (Figure 5B, pro*‐R* D‐transfer) . The observed stereospecificity is in full agreement with the relative position of NADH and *γ*‐butyrolactone in the active site of Cvir‐Hbd2 (Figure 5C).

### 2.9 Mössbauer and Electron Paramagnetic Resonance (EPR)‐Spectroscopic Analysis of FeADHs

**FIGURE 6:**
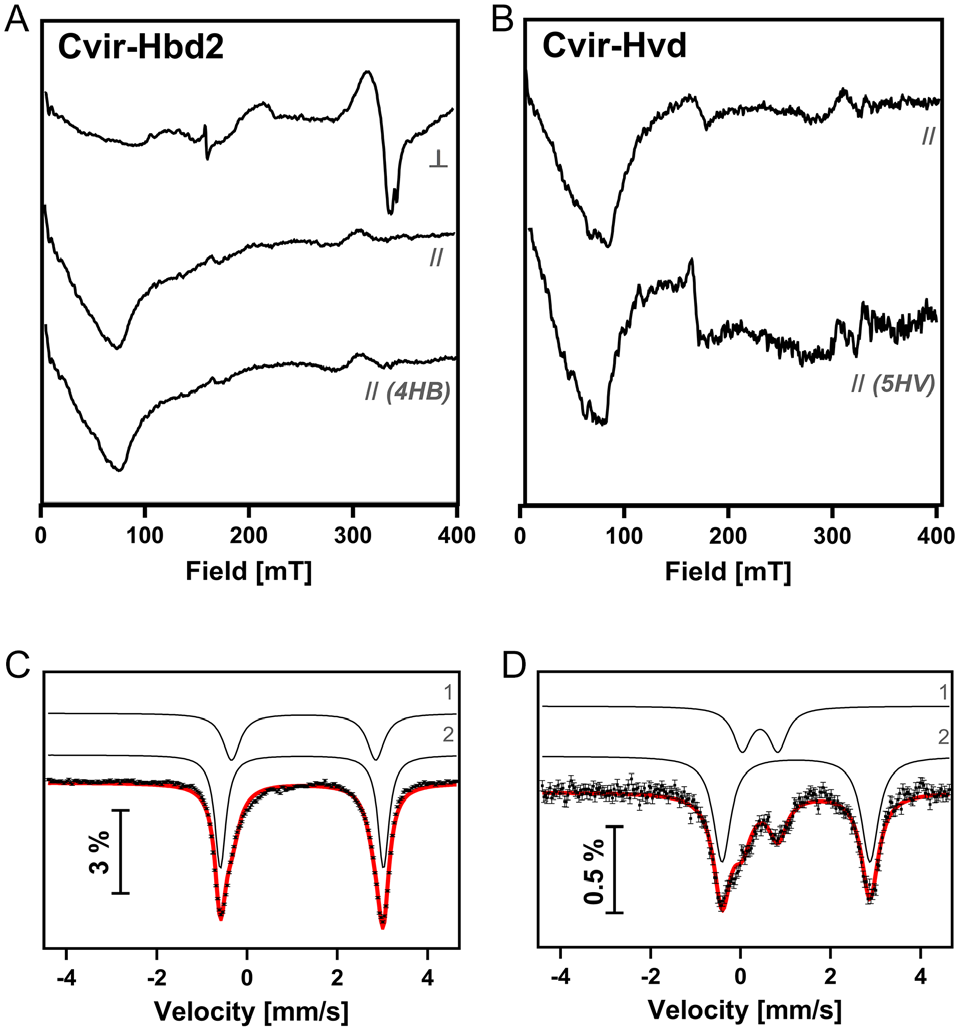
EPR and Mössbauer spectroscopy of the Fe^2+^ center in Cvir‐Hbd2 and Cvir‐Hvd. (A) Parallel (9.35 GHz) and perpendicular mode (9.62 GHz) EPR spectra of Cvir‐Hbd2 (~0.7 mM) before and after addition of 4‐hydroxybutyrate (10 mM) were recorded at 4.7 K, 0.8 mT modulation amplitude, 90 kHz modulation frequency, and 1024 points over 0–400 mT. (B) Parallel mode EPR spectrum of Cvir‐Hvd (~0.15 mM) before and after addition of sodium 5‐hydroxyvalerate (10 mM). EPR conditions as in A. (C,D) Mössbauer spectrum (no applied field, 77 K) of as isolated, ^57^Fe‐enriched Cvir‐Hbd2 (~2 mM) (C) and Cvir‐Hvd (~0.2 mM) (D).

Since scarce data on spectroscopy of FeADHs is available, EPR and Mössbauer spectroscopy were performed on as‐isolated enzymes to determine the oxidation and spin state. In the perpendicular mode X‐band EPR spectrum of Cvir‐Hbd2, a weak signal at *g *≈ 4.3 was observed, consistent with traces of high‐spin ferric ions, along with features at *g* = 2.02 and a very broad signal around *g *≈ 13. In parallel mode, the signals at *g *≈ 4.3 and 2.02 almost vanished, indicating that they arise from noninteger spin systems. In contrast, the signal at *g *≈ 13 intensified at least fivefold, typical for *S* = 2 ferrous ions [31, 32]. Considering the iron content and metal dependence of activity (Table 1), we conclude that the signal arises from ferrous ions in the active site. Addition of sodium 4HB did not alter the spectrum (Figure 6A). Similar results were obtained for Cvir‐Hvd, defining a high‐spin Fe^2+^ center alongside with a slightly higher Fe^3+^ contribution and lack of spectral changes upon substrate addition (Figure 6B).

For Mössbauer spectroscopy, both enzymes were expressed in *E. coli* grown on ^57^FeCl_3_‐supplemented minimal medium. The spectra of the purified FeADHs (77 K) showed enrichment of ^57^Fe without reconstitution. The spectrum of Cvir‐Hbd2 was fitted with two Fe^2+^ components (Area_1_ = 39%, *δ*_1_ = 1.25 mm/s, Δ*E*_Q,1_ = 3.19 mm/s, *Γ*_1_ = 0.46 mm/s; Area_2_ = 61%, *δ*_2_ = 1.22 mm/s, Δ*E*_Q,2_ = 3.61 mm/s, *Γ*_2_ = 0.30 mm/s). In agreement with EPR spectroscopic data, the isomer shift of ~1.23 mm/s measured for both components is consistent with high‐spin Fe^2+^ (Figure 6C) [33]. Differences in quadrupole splitting suggest species with variation in ligand field symmetry, potentially corresponding to forms of Cvir‐Hbd2 with or without NAD(H). Similar effects were observed for ^57^Fe‐reconstituted horse liver ADH (MDR‐superfamily) [34]. For Cvir‐Hvd, due to lower signal to noise, only one high‐spin ferrous component was fitted (Area_1_ = 70%, *δ*_1_ = 1.23 mm/s, Δ*E*_Q,1_ = 3.29 mm/s, *Γ*_1_ = 0.46 mm/s), which corresponds to the average of the two ferrous components in Cvir‐Hbd2. Contrarily, in this preparation, 30% of the area was fitted with parameters (*δ*_2_ = 0.43 mm/s, Δ*E*_Q,2_ = 0.79 mm/s, *Γ*_2_ = 0.48 mm/s) for a high‐spin ferric ion species, possibly arising from partial oxidation during sample preparation (Figure 6D).

## 3 Conclusion

This study provides a comprehensive characterization of FeADHs from *C. viride*, linking genomic information to enzymatic function. Of three 4HB dehydrogenases, one (Cvir‐Hbd2) was structurally and mechanistically analyzed, revealing key residues governing catalysis. A conserved tyrosine (Y256) plays a central role in substrate binding and activation as base. In addition, a conserved asparagine (N270) located in close proximity to the iron ion appears to modulate the electronic properties, balancing substrate affinity and catalytic turnover. Our data expand the aspartate or threonine signature for NAD^+^ specificity in the Rossmann fold to include asparagine (Figure S5) [4]. The hydride transfer from NADH for Cvir‐Hbd2 and Cvir‐Hvd was defined as pro‐*R*, demonstrating conservation of stereochemical course within the FeADH family [27, 28]. Three gene clusters encoding a transaminase, an ADH, and an acyl‐CoA dehydrogenase, as required for 5‐aminovalerate fermentation [19], were identified in the genome of *C. viride* (Figure 1). However, none of these three FeADHs are specific for 5HV. The absence of an obvious candidate for 5HV dehydrogenase in any other gene cluster of *C. viride* highlights that purification from the native source can be essential. We found Cvir‐Hvd to be encoded as a genomic singleton. This unexpected lack of pathway specific context suggests that other enzymes of 5‐aminovalerate fermentation may also be dispersed rather than organized in gene clusters. Enzyme characterization shows high activity for the physiological direction, while exhibiting activity toward a broad range of *ω*‐hydroxy acids, contrary to Cvir‐Hbd2. In the latter enzyme, R348 is positioned at the end of the substrate‐binding tunnel, where it could interact with the carboxylate moiety. Amino acid and structural alignment with Zmob‐AdhB, in which oxidized C362 has been suggested to contribute to ethanol binding [5], identifies this region of the active site as a substrate positioning determinant in FeADHs. In Cvir‐Hvd, the corresponding uncharged Asn360 may reflect its broader substrate profile (Figure S17). Substitution of active‐site Fe^2+^ with Mn^2+^ confers oxygen tolerance while maintaining comparable substrate affinity. Together with the exceptionally high activity toward C6 and even longer *ω*‐hydroxy acids, Cvir‐Hvd is a very attractive enzyme to insert into novel pathways for sustainable access to nylon‐6 precursors or polyketide synthases [12, 35, 36].

## 4 Experimental Section

### 4.1 General

Chemicals and reagents were purchased from Carl Roth, TCI, BLDPharm, and Sigma‐Aldrich and used without further purification. Genes were obtained from Twist Bioscience as codon‐optimized constructs, cloned into pET29b(+) (NdeI/XhoI). Vector DNA sequences can be found in Supporting Information. Original data were deposited to Zenodo (https://doi.org/10.5281/zenodo.21626235).

### 4.2 Bioinformatic Analysis

*C. viride* DSM 6836 protein sequences from IMG/M were used for LC–MS data analysis and candidate gene identification [23]. Sequence alignments were performed using Clustal Omega. Structural models and experimental structures were visualized using PyMOL and ChimeraX.

### 4.3 Site‐Directed Mutagenesis

Site‐directed mutagenesis was performed by a two‐step PCR according to manufacturers’ instructions for Q5 polymerase. The first PCR was carried out using either the forward or reverse primer (Table S2) for eight cycles. In the second step, both reactions were combined, supplemented with fresh Q5 polymerase and amplified for 18 cycles. After DpnI treatment and transformation into *E. coli* NEB10 cells, plasmid DNA was isolated and Sanger sequenced.

### 4.4 Expression and Purification of Heterologously Expressed FeADHs

Plasmids were transformed into *E. coli* BL21 cells. Overnight precultures were grown in lysogeny broth (LB, pH 7) supplemented with 30 μg/mL kanamycin at 37 °C and 150 rpm.

For expression, 2 L LB in 5 L Erlenmeyer flasks were inoculated (1% v/v) and grown to OD_600 __nm_ = 0.3, cooled to 30 °C, and further incubated to OD_600__nm_ = 0.5. For ^57^Fe‐enrichment, cells were grown in M9 minimal medium supplemented with ^57^FeCl_3_ (25 µM) (Supporting Information). Expression was induced with 0.5 mM IPTG, followed by incubation for 16–18 h at 30 °C. Harvested cells were washed with BN buffer (50 mM BisTris, pH 7.5, 150 mM NaCl), and stored at −80 °C.

For purification, cell pellets were transferred to an anaerobic chamber (Coy Laboratory Products, O_2_ < 10 ppm and 5% H_2_), resuspended in BNT (BN buffer with 2 mM TCEP) supplemented with DNase (2 mg) and 4‐(2‐aminoethyl)benzenesulfonyl fluoride (0.1 mM), and lysed by sonication. After ultracentrifugation and filtration (0.45 µm), proteins were purified using Cytiva ProteinSelect resin (3 mL) equilibrated with BNT. Lysate was loaded (≈5 mL/min), followed by washing (15 mL BNT), incubation at 25 °C (2–18 h) and elution with 4 mL BNT. Aliquots in gas‐tight vials were flash‐frozen in liquid nitrogen, and stored at −80 °C. Nickel‐NTA purification was according to manufacturers instructions.

Protein characterization was carried out by SDS‐PAGE using Coomassie staining and Bradford assay (BioRad) with bovine serum albumin as standard. Protein‐bound iron was quantified as described previously [37]. NAD(H) content was determined spectrophotometrically at 260 and 340 nm corrected for protein absorbance from calculated extinction coefficients [38, 39, 40]. Size‐exclusion chromatography (ÄKTA Start) was performed on a HiPrep 26/60 Sephacryl S‐200 HR column at 2 mL/min BN.

### 4.5 Growth of *C. viride* and Identification of 5HV Dehydrogenase

*C. viride* (DSM 6836) obtained from DSMZ was grown (37 °C, 150 rpm, 24 h) in 5‐aminovalerate medium (Supporting Information), harvested by centrifugation, and stored at −80 °C. Two grams of cells were resuspended in buffer A (20 mM BisTris/HCl, pH 7; 25 mL) supplemented with protease inhibitor (Pierce) and DNase I (3 mg) and lysed by French press. After ultracentrifugation, supernatant was applied to a HiScreen Q FF IEX column (CV = 4.7 mL) equilibrated with buffer A, washed (5 CV), and eluted with a 20 CV linear gradient to buffer B (buffer A with 200 mM (NH_4_)_2_SO_4_). Cvir‐Hvd was identified by absorbance at 492 nm (10 mM sodium 5HV, 0.5 mM NAD^+^, 25 µM Meldola Blue, 0.5 mM INT, 10 mM semicarbazide, 100 mM Tris/HCl pH 8.0, 0.2% (w/v) Triton X‐100), pooled and adjusted to 2 M (NH_4_)_2_SO_4_. After centrifugation, the supernatant was applied to a HiScreen Phenyl Low Sub HIC column (CV = 4.7 mL) equilibrated with buffer C (buffer A with 2 M (NH_4_)_2_SO_4_), washed, and eluted with a 20 CV gradient to buffer A in 20 CV. Active fractions were pooled, aliquoted, flash‐frozen, and stored at −80 °C. Proteins were identified by SDS–PAGE, in gel digestion, LC–MS/MS, and MaxQuant analysis against the *C. viride* IMG/M proteome (Figure S18).

### 4.6 Crystallography of Cvir‐Hbd2

Cvir‐Hbd2 was eluted from CytivaProtein Select after 16 h incubation and reconstituted with 1 mM (NH_4_)_2_Fe(SO_4_)_2_ (15 min). After size‐exclusion chromatography in BNT, protein was concentrated to 10.8 mg/mL (ThermoScientific MWCO 30K). Aliquots (80 µL) were flash‐frozen under anaerobic conditions and stored at −80 °C.

Protein crystallization was performed under anoxic conditions as described for protein purification. Before crystallization, NADH (5.7 µL, 140 mM) and sodium 4HB (1.6 µL, 1 M) were added to the protein and incubated (45 min, 20 °C). Crystallization was performed using the sitting‐drop vapor diffusion method (18–20 °C) by mixing equal volumes of protein and reservoir solution (0.08 M MgSO_4_, 0.02 M NaCl, 0.02 M 2‐(*N*‐morpholino)ethanesulfonic acid, 10% (w/v) PEG 1450 (pH 6); condition A3 from screen MemGold‐2, Molecular Dimension Ltd., UK). Crystals appeared within 40 h and were cryoprotected in the reservoir solution supplemented with 15% (v/v) (2*R*, 3*R*)‐butanediol before flash‐cooling in liquid nitrogen. Data collection and analysis are described in the Supporting Information. The coordinates and structure factors amplitudes of Cvir‐Hbd2 are available at the Protein Data Bank (PDB, www.pdb.org) under accession number 30SM. Replacement of the native N‐terminal methionine with a KFE sequence, introduced for restriction site generation and optimized cleavage of the Cytiva ProteinSelect tag, adds two residues to Cvir‐Hbd2. Accordingly, residue numbers in the PDB file are shifted by +2 relative to the native sequence.

### 4.7 Measurement of Enzyme Activity

All steps were performed under anaerobic conditions. Enzyme solutions were diluted to 1 mg/mL in BNT and reconstituted with 1 mM (NH_4_)_2_Fe(SO_4_)_2_ (25 °C, 60 min). Reactions were initiated mixing 20 µL of diluted enzyme to give Δ*A*_340 nm_/min ≈ 0.1–0.5 and 980 µL buffer containing the substrate and NAD(H) in a 1 cm cuvette and monitored for 15 s. 5OV was added immediately prior to measurement. For EDTA inactivation, enzyme (0.3–3 mg/mL) was incubated for 16 h at 25 °C. For metal ion reconstitution, enzyme was incubated with 10 mM EDTA (16 h, 25 °C), desalted using a PD‐10 column, and subsequently incubated with 1 mM of divalent metal chloride (1 h). Enzyme activity is expressed in units (U), where 1 U corresponds to 1 µmol of substrate converted per minute.

### 4.8 Stereospecificity of Hydride Transfer

NADD ([4*R*‐^2^H]NADH) was generated enzymatically from NAD^+^ using deuterated ethanol and yeast ADH1 and purified via anion‐exchange chromatography (Supporting Information). The product was dissolved in D_2_O‐based 100 mM potassium phosphate buffer (pH* = 6.3).

Mixtures containing NADH or NADD, buffer, 5OV, and enzyme were incubated until a stable 340 nm reading, filtered (30 kDa cutoff), supplemented with d_4_‐TMSP (1 mM), and analyzed by ^1^H NMR.

### 4.9 Spectroscopy

Spectra were recorded using a UV‐1900i UV–Vis spectrometer (Shimadzu) and a Chirascan V100 CD spectrometer (Applied Photophysics) in 1 cm quartz cuvettes.

NMR spectra of samples (500 µL) in 5 mm NMR tubes were recorded on Bruker AVANCE 400 or 600 MHz spectrometers. Data were processed using MestReNova (v14.2.3) with automatic phase and baseline correction; chemical shifts were referenced to d_4_‐TMSP or residual CHCl_3_.

EPR spectra were recorded with a Bruker Elexsys E580 X band spectrometer with a dual‐mode resonator (ER4116) and an Oxford Instruments ESR900 helium flow cryostat connected to a stinger (Cold Edge Technologies).

Mössbauer samples were measured and analyzed as previously [41].

### 4.10 Statistical Analysis

Enzyme activity measurements, iron and protein quantification were performed as technical duplicates. Reported values represent the mean of two independent protein purifications. Error bars correspond to the standard error of the mean (SEM). For kinetic analyses, specific activities from biological duplicates were normalized to the mean prior to fitting. Data analysis and fitting were performed using Excel and GraphPad Prism (v11.0.1). Kinetic parameters were determined by nonlinear regression using the substrate inhibition model in GraphPad.

## Funding

This study was supported by the Deutsche Forschungsgemeinschaft (311771384, 444947649, 397427223).

## Conflicts of Interest

The authors declare no conflicts of interest.

## Supporting information

The authors have cited additional references within the Supporting Information [42, 43, 44, 45, 46, 47].

## Data Availability

The data that support the findings of this study are openly available in Zenodo at https://doi.org/10.5281/zenodo.21626235.
