# Supplementary material for: ω‐Hydroxyacid Dehydrogenases From Clostridium viride: Iron‐Containing Biocatalysts With Biotechnological Potential
Source: Chembiochem. 2026 Aug 21;27(16):e70511. doi: 10.1002/cbic.70511 (PMC13495136; doi:10.1002/cbic.70511)
Supplement: Supplementary file 1 [file CBIC-27-e70511-s001.pdf]

Synthesis of Methyl-5-hydroxyvalerate

δ-Valerolactone (150 mmol, 15 g) was dissolved in methanol (100 mL), and triethylamine (52 mmol, 7.2 mL) was added. The mixture was stirred at room temperature for 24 h and the solvent removed under reduced pressure, yielding the product as a colorless oil in stochiometric amounts with approximately 5% residual educt.

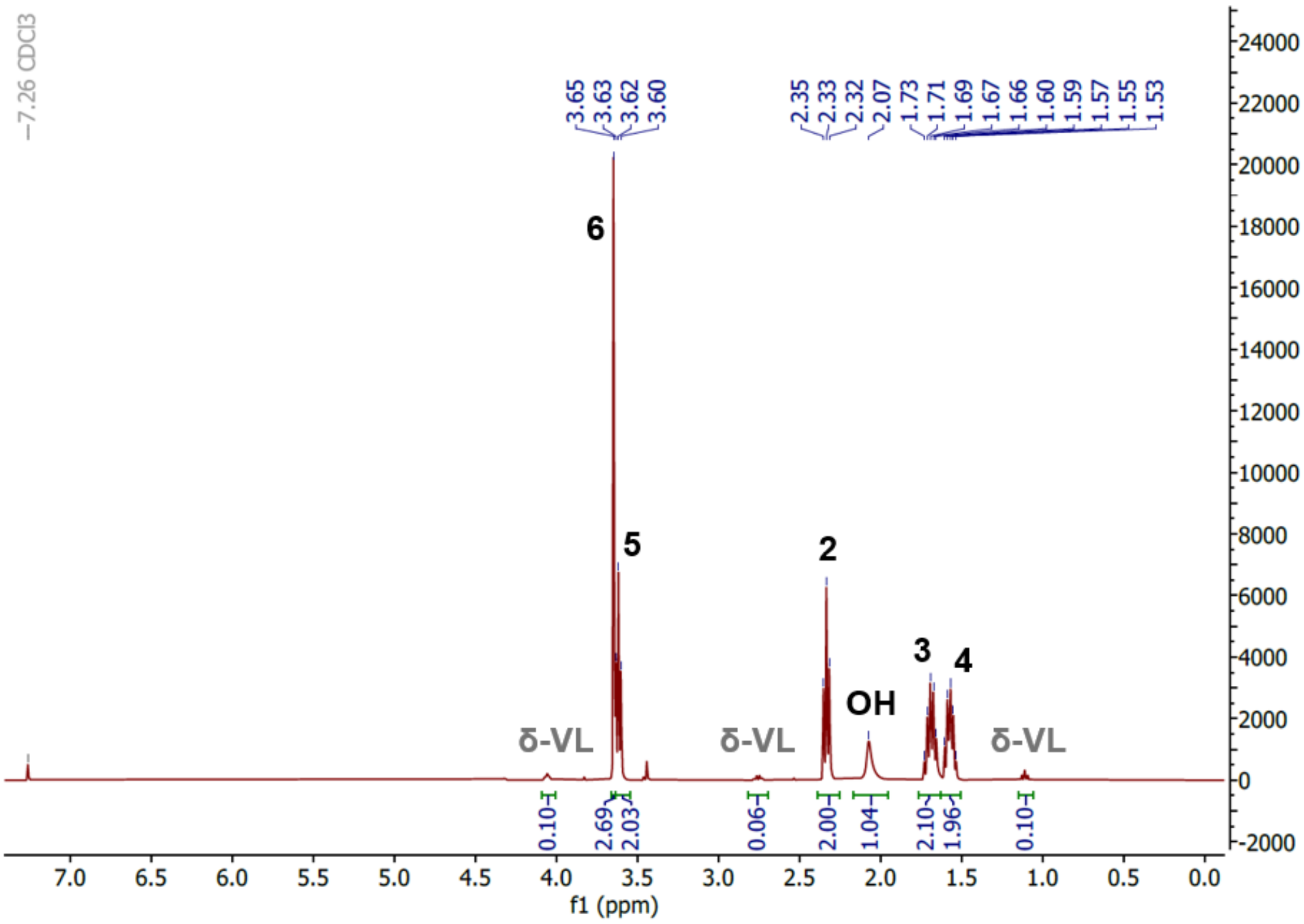

^{1}H NMR (400 MHz, $CDCl_3$) δ 3.65 (s, 3H, $-CH_3$), 3.62 (t, 2H, 6.4 Hz, H-5), 2.34 (t, 2H, 7.2 Hz, H-2), 2.07 (1H, -OH), 1.70 (m, 2H, H-3), 1.57 (m, 2H, H-4).

Synthesis of Methyl-5-oxovalerate

Methyl 5-oxovalerate was prepared by Swern oxidation. Oxalyl chloride (130 mmol, 11.1 mL) in dry dichloromethane (250 mL) was cooled to -80 °C, followed by dropwise addition of DMSO (250 mmol, 17.8 mL) in dichloromethane (250 mL). After 30 min, a solution of methyl 5-hydroxyvalerate (110 mmol, 14.5 g) in dichloromethane (250 mL) was added and stirred for 60 min at -80 °C. Triethylamine (50 mL) was added, and the reaction was warmed to room temperature over 90 min. The reaction was quenched with water, and the organic phase was separated, dried over $Na_{2y}SO_4$, and the solvent removed under reduced pressure. The crude product was purified by silica gel chromatography (EtOAc/cyclohexane 3:2 (*v/v*)) to yield methyl 5-oxovalerate (6.1 g, 46 mmol, 43 % yield) as a pale yellow oil with traces of solvent that were used for chromatography. The oil was stored for months at -20 °C without notable decomposition.

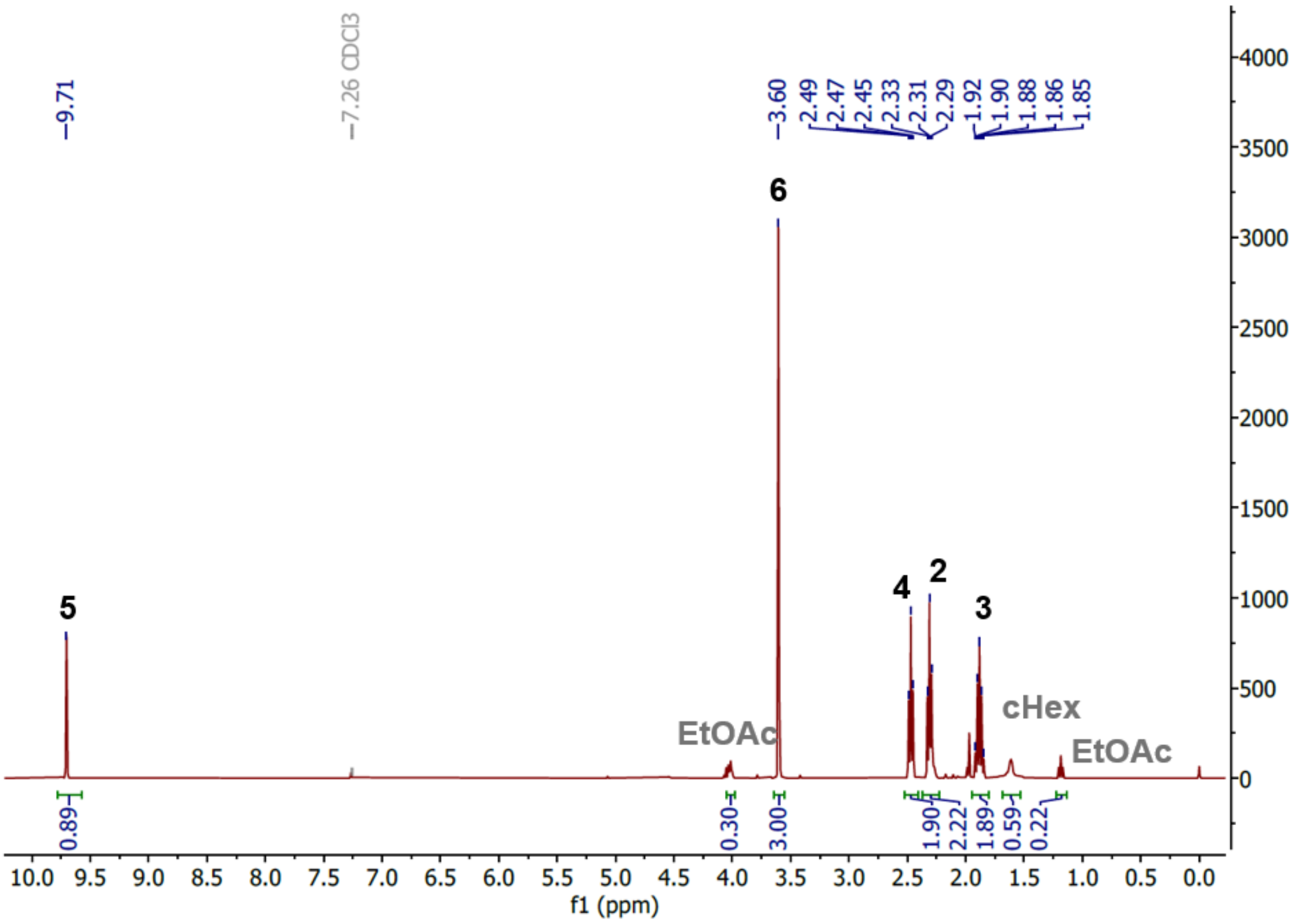

^{1}H NMR (400 MHz, $CDCl_3$) *δ* 9.71 (s, 1H, H-5), 3.60 (s, 3H, $-CH_3$), 2.47 (t, 2H, 7.2 Hz, H-4), 2.31 (t, 2H, 7.3 Hz, H-2), 1.88 (q, 2H, 7.2 Hz, H-3).

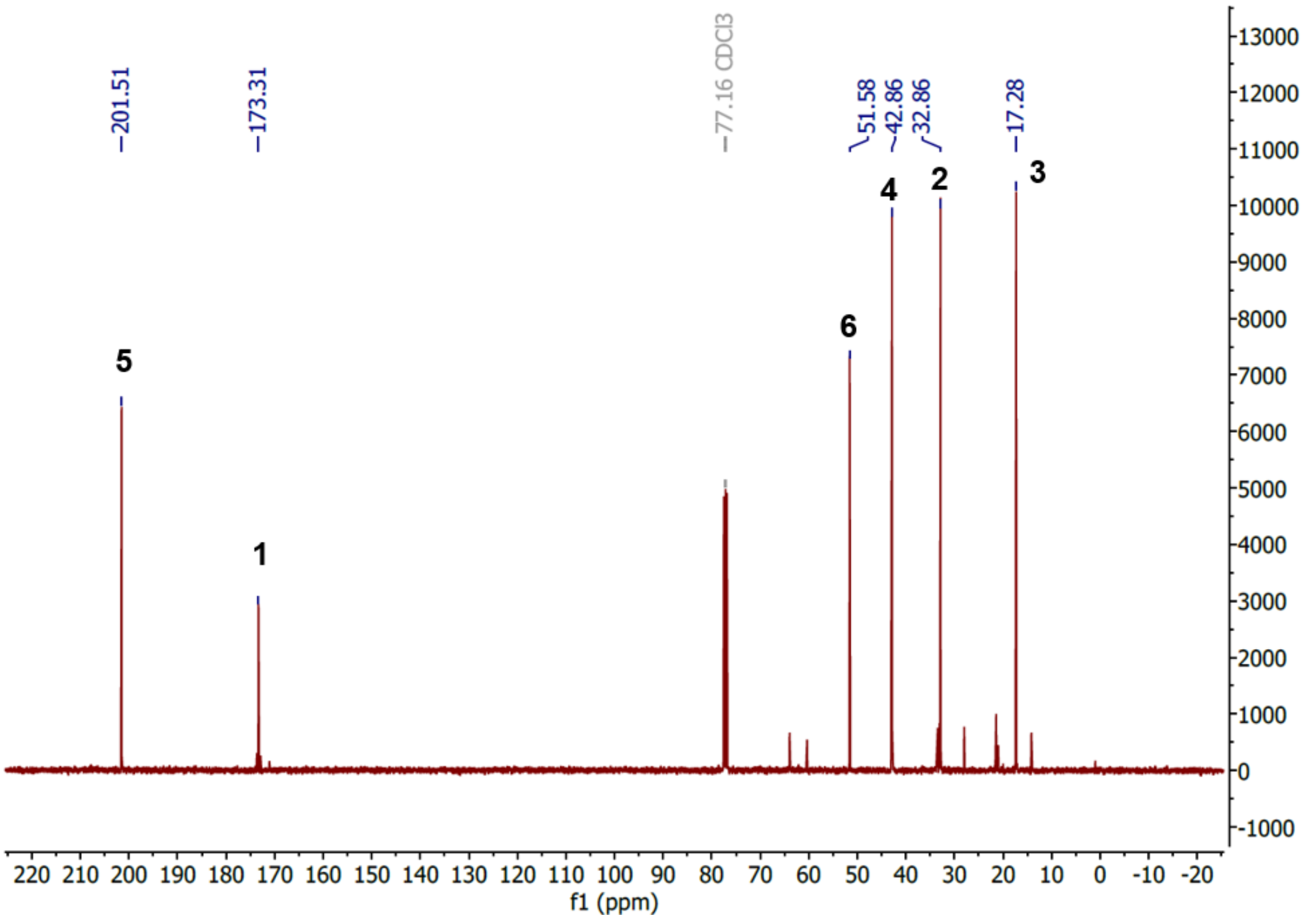

^{13}C NMR (100 MHz, $CDCl_3$) *δ* 201.51 (s, C-5), 173.31 (s, C-1), 51.58 (s, $-CH_3$), 42.86 (s, C-4), 32.86 (s, C-2), 17.28 (s, C-3).

Synthesis of 5-Oxovaleric acid

5-Oxovaleric acid was obtained by acidic hydrolysis of methyl 5-oxovalerate (with minor residual solvent; 2 g, 15 mmol) in aqueous 1 M HCl at room temperature for 24 h. The mixture was saturated with NaCl, extracted with diethyl ether (3 x 50 mL), dried over Na_2SO_4, and concentrated under reduced pressure. The product was purified by silica gel chromatography to yield 5-oxovaleric acid as pale yellow oil (200 mg, 1.8 mmol, 12 % yield). Though the ^{1}H-NMR spectrum indicated several minor impurities, the lability of the substance required that no further purification steps would be undertaken. Therefore, the product was suspended in water, adjusted to pH 6.5 with NaOH to dissolve the oil, aliquoted, and stored at -80 °C. The actual concentration of 5-oxovalerate in the preparation was determined enzymatically by reacting the stock solution with an approximate 2-fold excess of NADH under anaerobic conditions with Cvir-Hvd in 100 mM BisTris/HCl pH 6 and monitoring NADH consumption via the absorbance at 340 nm.

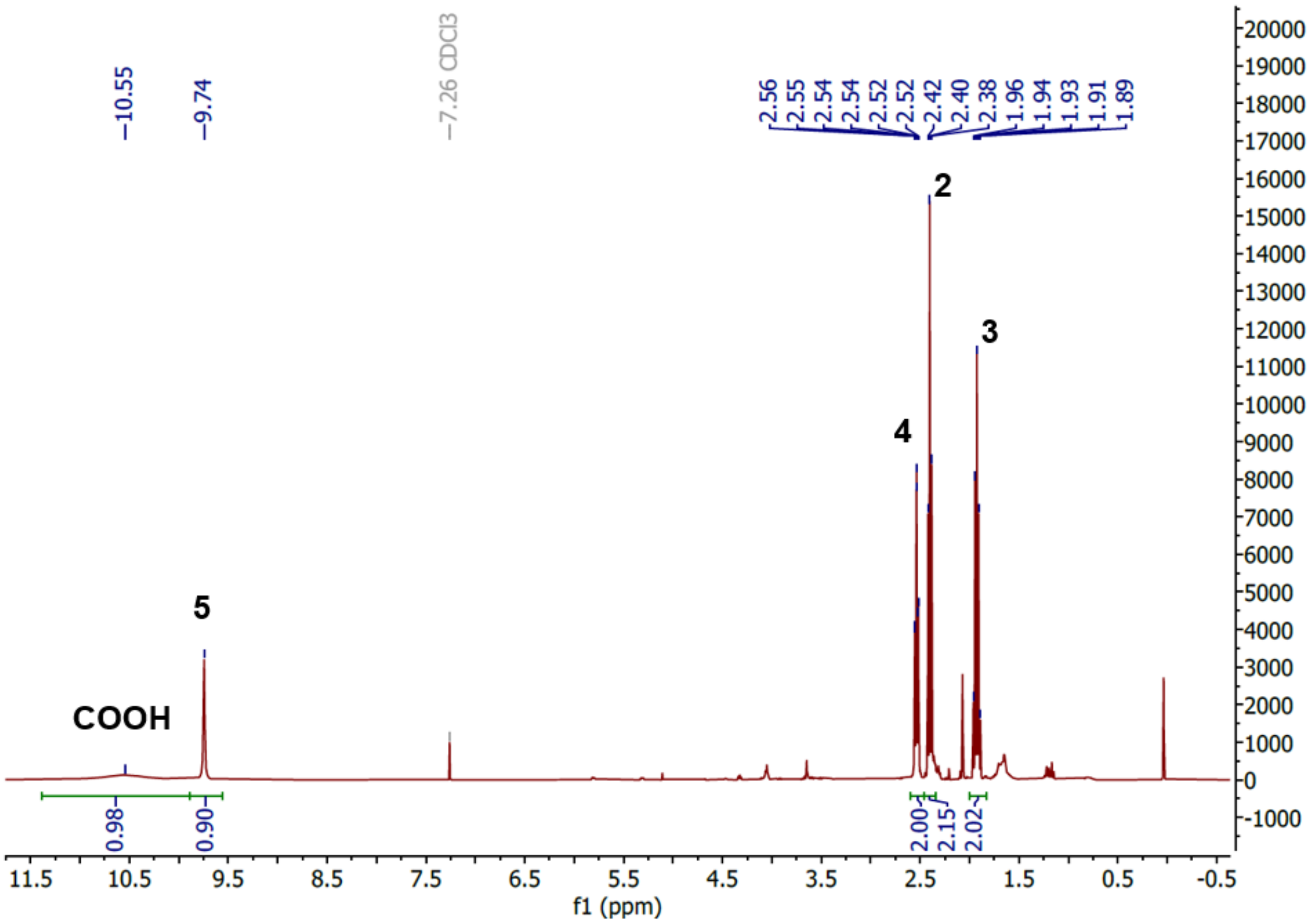

^{1}H NMR (400 MHz, $CDCl_3$) δ 10.55 (1H, -COOH), 9.74 (s, 1H, H-5), 2.54 (td, 2H, 7.2 Hz, 1 Hz, H-4), 2.40 (t, 2H, 7.4 Hz, H-2), 1.93 (q, 2H, 7.3 Hz, H-3).

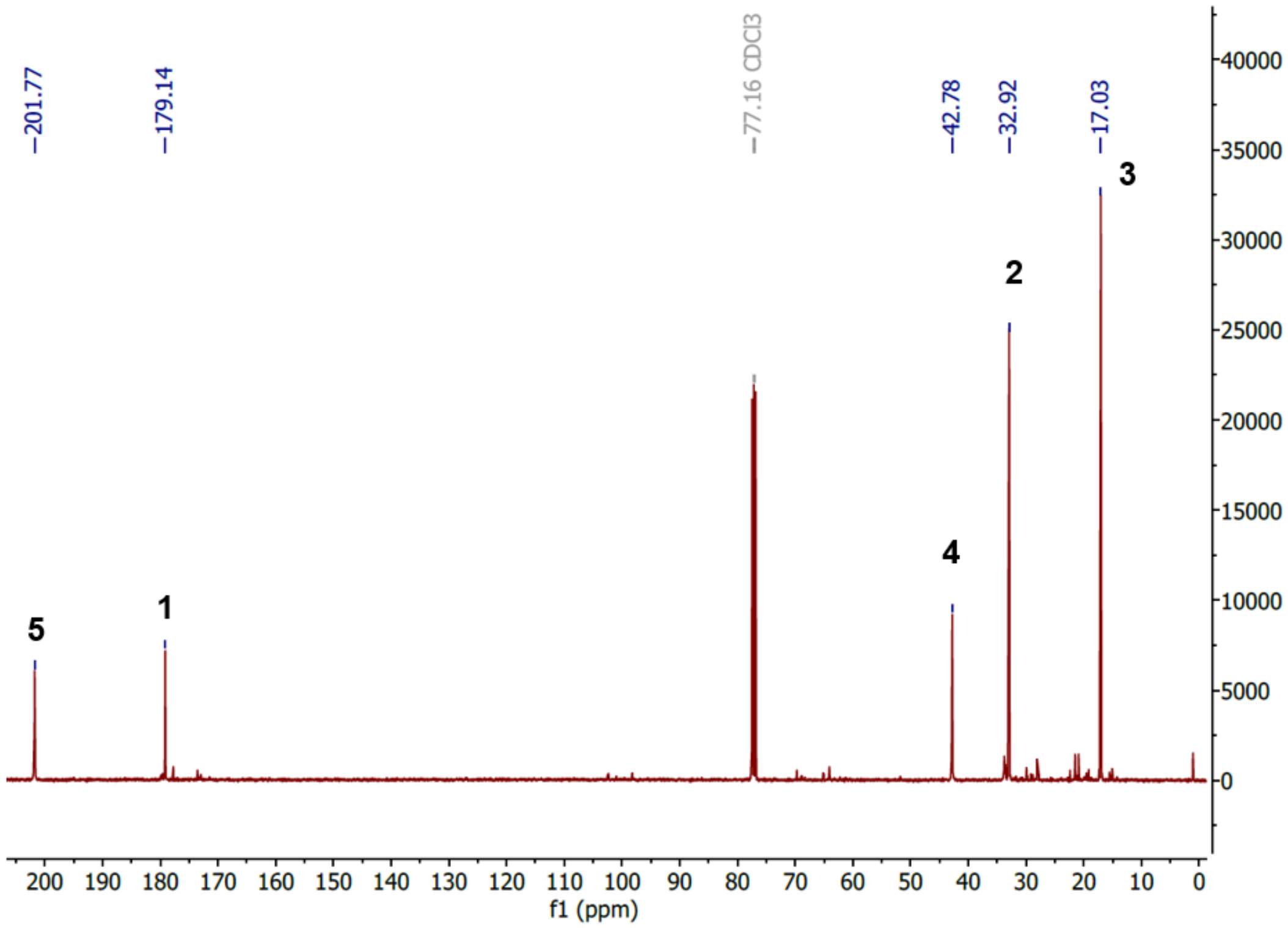

^{13}C NMR (100 MHz, $CDCl_3$) *δ* 201.77 (s, C-5), 179.14 (s, C-1), 42.78 (s, C-4), 32.92 (s, C-2), 17.03 (s, C-3).

Synthesis of NADD

NADD ([4*R*-^{2}H]NADH) was generated enzymatically by reduction of NAD^+ (26 mg, 40 µmol) dissolved in 8 mL TAPS/NaOH buffer (25 mM, pH 9.0) using 25 µL d_6-ethanol and 1 mg of *S. cerevisiae* ADH1 (Sigma-Aldrich). The reaction was incubated at 37 °C for 30 min and subsequently purified by anion exchange chromatography (HiScreen Q FF, ÄKTA Start) using 50 mM ammonium bicarbonate for equilibration and washing. NADD was eluted with 200 mM ammonium bicarbonate, and fractions absorbing at 340 nm were collected.

The product was concentrated under reduced pressure (40 °C, 1 mbar) and dissolved in D_2O-based potassium phosphate buffer (100 mM, pH* 6.3) and adjusted to pH* 6.3 with DCl. pH* equals the direct readout from the H_2O calibrated pH-electrode, without adjustment to D_2O.

For the determination of stereospecificity, 5-oxovalerate solution (25 µL, 170 mM) and 10 µL of enzyme (~ 5 mg/mL) were added to 2 mM solution of NADD in 100 mM KP_i in D_2O (pH* = 6.3). After incubation (5–30 min, depending on enzyme), samples were filtered using 30 kDa cutoff centrifugation concentrators to remove protein. The filtrate was supplemented with d_4-TMSP (1 mM) and analyzed by ^{1}H NMR (600 MHz, water suppression, 128 scans).

Preparation of *Clostidium viride* medium

5-Aminovalerate-containing medium was prepared by dissolving δ-valerolactam (5.0 g), KOH (6.6 g), and NaOH (1.2 g) in ddH_2O (100 mL). The solution was incubated at 25 °C for 24 h and then diluted with ddH_2O to 800 mL. The pH was adjusted to 8.0 with H_3PO_4 (85 % (*w/v*)). Activated charcoal (5.0 g) was added, and the suspension was stirred for 10 min before filtration through a glass fiber filter. Yeast extract (7.5 g), VR mix (4.0 mL, according to DSMZ medium 526), and resazurin (0.5 mL, 0.1% (*w/v*)) were added to the filtrate. If necessary, the pH was adjusted to 7.8 with KOH or H_3PO_4, and the final volume was brought to 1 L with ddH_2O.

A Duran glass bottle was filled with an appropriate volume of the 5-aminovalerate medium, and the solution was sparged with nitrogen for 5 min before the bottle was sealed with a rubber septum. The headspace was exchanged three times with nitrogen at 1.2 bar, and the bottle was then autoclaved. Prior to inoculation with the lyophylized DSMZ culture of *C. viride*, or a preculture, 10 mL/L of a sterile-filtered, anaerobic *L*-cysteine solution (160 mM, pH 7.8) was added and incubated (37 °C) until resazurin became colorless.

Preparation of ^{57}Fe-supplemented minimal medium

The M9 salts solution (5 x) contained 37.6 g Na_2HPO_4 * $2H_2O$, 30.7 g KH_2PO_4 * $7H_2O$, 2.5 g NaCl, and 5.0 g NH_4Cl per liter of ddH_2O. For 1 L medium, 200 mL M9 salts (5 x), 20 mL 20% *w/v* glucose, 1 mL 1 M $MgSO_4$, 0.1 mL 1 M $CaCl_2$, and 0.5 mL 50 mM $^{57}FeCl_3$ with the required antibiotic were combined and adjusted to 1 L with ddH_2O.

In Gel Digest, Peptide purification and MS/MS analysis

Protein (2 µg/lane) was prepared for SDS-PAGE and loaded. Electrophoresis was performed at 75 V for 20 min, followed by 150 V until the bromophenol blue front entered approximately 0.5 cm into the running gel (~ 5 min). After electrophoresis, the stacking gel was removed, and the upper part of the resolving gel containing the proteins was excised and cut into pieces of approximately 1 × 1 mm. The gel pieces were transferred to LoBind microcentrifuge tubes.

The following steps were carried out at room temperature, except if stated otherwise. Gel pieces were incubated with 1 mL fixation solution consisting of 40% (*v/v*) ethanol and 7% (*v/v*) acetic acid (15 min, 400 rpm), washed with 1 mL water (15 min, 400 rpm), and subsequently washed with 1 mL washing buffer consisting of 25 mM ammonium bicarbonate (ABC) and 50% (*v/v*) ethanol (20 min, 400 rpm). The gel pieces were then dehydrated twice with 1 mL acetonitrile (20 min, 400 rpm). Between each step, samples were centrifuged for 1 min at 900 xg, and the supernatant was removed. The gel pieces were dried in a SpeedVac at 45 °C for 5 min.

For reduction, the dried gel pieces were rehydrated in 1 mL reduction buffer consisting of 10 mM DTT and 50 mM ABC and incubated (55 °C, 1 h). The supernatant was removed, and 0.2 mL alkylation buffer consisting of 55 mM 2-chloroacetamide and 50 mM ABC was added (1.5 h). The gel pieces were washed twice with 1 mL solution of 50 mM ABC (20 min, 400 rpm), dehydrated twice with 1 mL of acetonitrile (20 min, 400 rpm), and dried in a SpeedVac at 45 °C for 5 min.

For proteolytic digestion, the gel pieces were rehydrated with 0.2 mL trypsin solution (12.5 ng/µL trypsin in 50 mM ABC) at 4 °C for 20 min. The trypsin solution was removed, 50 mM ABC was added (0.2 mL), and digestion was carried out at 37 °C for 16 h. The digestion was stopped by addition of trifluoroacetic acid (TFA; 2 µL), and the supernatant was transferred to a fresh LoBind microcentrifuge tube. The gel pieces were washed once with 150 µL 50 mM ABC and the peptides extracted twice with each 150 µL extraction buffer consisting of 3% (*v/v*) TFA and 30% (*v/v*) acetonitrile. The combined supernatants were concentrated in a SpeedVac at 45 °C to a final volume of 200 µL. Prior to StageTip purification, samples were acidified to approximately pH 2 using TFA (10% (*v/v*)).

StageTips were prepared using three layers of SDB-RPS material (3M, cat. no. 2241). The StageTips were activated with acetonitrile (100 µL), equilibrated with 30% (*v/v*) methanol/1% (*v/v*) TFA (100 µL), and subsequently washed with 0.2% (*v/v*) TFA (100 µL). Acidified peptide samples were loaded onto the StageTips, which were then washed three times with 100 µL of 0.2% (*v/v*) TFA. Peptides were eluted with 80% (*v/v*) acetonitrile/ 5% (*w/v*) NH_3 (60 µL) and dried in a SpeedVac at 25 °C for 40 min. For LC-MS/MS analysis, dried peptides were dissolved in a buffer consisting of 0.1% (*v/v*) formic acid and 0.01% (*v/v*) TFA (12 µL) by sonication in an ultrasonic bath for 5 min. A sample volume of 4 µL was used for MS/MS analysis.

Peptides were separated using an Easy-nLC 1200 system (Thermo Scientific) coupled via a Nanospray Flex ion source to a Q Exactive HF mass spectrometer. Separation was performed on an analytical column with a length of 50 cm and an inner diameter of 75 µm (New Objective), packed in-house with C18 resin (ReproSil-Pur 120, 1.9 µm; Dr. Maisch). The column was operated at a constant flow rate of 250 nL/min. Peptides from in gel digests were separated using a 45 min gradient from aqueous 0.1% (*v/v*) formic acid to 80% acetonitrile with 0.1% (*v/v*) formic acid.

Peptides were analyzed in positive-ion mode using a spray voltage of 2.3 kV and a capillary temperature of 320 °C. MS spectra were acquired in profile mode over a mass range of 300–1650 m/z at a resolution of 60000, with a maximum injection time of 20 ms or a maximum of 3×10^6 ions using automatic gain control (AGC). Fragmentation was triggered for the top 15 precursor ions with charge states of 2–8 in a data-dependent acquisition mode, using a dynamic exclusion window of 20 s. Precursors were isolated with a 1.4 m/z window, and MS/MS spectra were acquired in profile mode at a resolution of 15000, with a maximum injection time of 80 ms and an AGC target of $1 * 10^5$ ions.

Data collection and analysis for protein crystallography

Diffraction data at wavelengths of 0.9813 Å and 1.735984 Å (Fe edge) were collected at 100 K at beamline BL 14.1 operated by the Helmholtz-Zentrum at the BESSY II electron storage ring (Berlin, Germany)[46] and were integrated and scaled using DSAPP.[45] The crystals belonged to $P6_3$ space group with molecular weight of 80 kDa per asymmetric unit and a solvent content of 58 %. The intial phases were obtained using Patterson search techniques with *phaser*[47] as implemented in Phenix.[43] The search model was a Cvir-Hbd2 model calculated using AlphaFold3 (https://alphafoldserver.com).[42] The iron ion, NADH, and γ-butyrolactone were modelled only when their positions were clearly shown on the electron density map. Iterative cycles of manual model building in Coot[44] and refinement using reflections up to a resolution of 1.73 Å in Phenix (*phenix.refine*)[43] resulted in a protein model with good statistics and stereochemical values (R_{work}/R_{free} = 0.177/0.199). **Table S3** shows the statistics for data collection and protein model refinement. The molecular interface of the dimer was analyzed using the "Proteins, Interfaces, Structures and Assemblies server" (PDBePISA, https://www.ebi.ac.uk/pdbe/pisa/).

Table S1. Kinetic parameters of Cvir-Hvd with ω-hydroxy carboxylates of different chain lengths. Apparent K_m and V_{max} values were estimated for C4–C12 ω-hydroxycarboxylic acids at 1 mM NAD^+ in 100 mM Tris/HCl, pH 9.0. Activities were measured with 1, 2, 5, 10, and 20 mM substrate, and k_{cat}/K_m values were calculated from the derived kinetic parameters. Due to the very low activity with 4HB, the corresponding values were estimated. The values for 5HV were taken from more detailed kinetic measurements shown in main section and are included here for comparison. Data are duplicates for one protein preparation.

Chain length	K_m (mM)	V_{max} (U/mg)	k_{cat}/K_m (s^{-1} M^{-1})
4	~15	~ 1	< 50
5	0.4	150	$2.6 * 10^5$
6	7.1	122	$1.2 * 10^4$
7	4.1	78	$1.3 * 10^4$
8	12	16	$0.9 * 10^3$
9	22	125	$3.9 * 10^3$
10	15	80	$3.6 * 10^3$
12	5.7	37	$4.4 * 10^3$

Table S2. Primers used for mutagenesis

Hbd2-N270A-for	GCCGCCTACGCAATGTTCACTGGCGTTTTGAAGAAGTACC
Hbd2-N270A-rev	CGTAGGCGGCCTCTCCGTGTGGTACGTGG
Hbd2-Y256A-for	CATTAAGCGCTCCATTAGGTGCGGTATACCACG
Hbd2-Y256A-rev	CTAATGGAGCGCTTAATGCGTGGACTGCTGC
Hbd2-R348A-for	CCAAACTGCTCTCATGGCTAATAACTTCGTTCC
Hbd2-R348A-rev	CATGAGAGCAGTTTGGTTCTCTCGAACGG
Hbd2-R348E-for	CCAAACTGAGCTCATGGCTAATAACTTCGTTCC
Hbd2-R348E-rev	CATGAGCTCAGTTTGGTTCTCTCGAACGG
Hvd4-D39G-for	CTTATAAGTGGCCGAGGTCTTGAGGCAATCGG
Hvd4-D39G-rev	CCTCGGCCACTTATAAGAAGCACATTGTCACTACC
Hvd4-D194A-H198A-for	GGTATTGCCGCCTTCATAGCCGCTCTCGAAGCATATATCAATACTGC
Hvd4-D194A-H198A-rev	GAGCGGCTATGAAGGCGGCAATACCACAAGCGGCTGCGATTGAAGG

Table S3. Crystallographic data collection and refinement statistics. Values in parentheses are given for the highest resolution shell. Friedel pairs are treated as different reflections.

**PDB-ID (extended)**	30SM (PDB ID pdb_000030SM)
**Data collection**	
Source, wavelength (Å)	BL 14.1 BESSY II, 0.9184
Resolution (outer shell) (Å)	41.85 - 1.73 (1.83 - 1.73)
Space group	$P6_3$
Cell constants (a,b,c (Å), α,β,γ (°))	116.66, 116.66, 120.15, 90, 90, 120
R_{meas}	0.227 (1.989)
R_{pim}	0.071 (0.613)
Total, unique reflections	1977761 (321781), 190957 (30832)
Mean(I)/sd(I)	11.12 (1.31)
CC(1/2)	1.00 (0.25)
Completeness (%) & Multiplicity	100 (100) & 10.4 (10.4)
**Refinement**	
Reflections used (total/free set)	96509/2099
R_{work} , R_{free} factor	0.176 (0.318), 0.199 (0.351)
Average B (Å^2) (overall/protein/water/NAD/GBL)	27.18 / 25.72 / 38.05 / 24.31 / 38.57
Average B (Å^2) (main/side chain A & main/side chain B)	22.64/26.08 & 25.34/29.00
Ramachandran-statistics (%) favored, allowed, disfavored	98.51, 1.09, 0.41
RMS-deviation from ideal geometry bonds (Å), angles (°)	0.007, 0.90

```
Zmob-AdhB    NDPLLMVGMPKGLTAATGMDALTHAFEAYSST-AATPITDACALKAASMIAKNLKTACDN    233
Cnec-Hbd     CDPELTLGLPAGLTAATGMDAIAHCIETFLAP-AFNPPADGIALDGLERGWGHIERATRD    232
Cklu-Hbd     LVPEFIKGLPYKFFVTSSVDALIHATEAYVSP-NANPYTDMFSVKAMELILNGYMQMVEK    221
Cvir-Hbd1    LIPELLARLPFRFFATSSIDALIHAIESTLSP-KASEITQLFSYKAIELILNGYLEIRDQ    220
Cvir-Hbd2    LIPELLEGLPFRFFATSSIDALVHAVESSLSP-KASDLSKLFGYRAIDMILRGFLTIRDQ    220
Cvir-Hbd3    LIPALLMGLPFRFFATSAIDALVHAVESSLSP-KASAASKLFSYQAIDMILRGFLEIRNQ    220
Cvir-Hvd     LDPELIMTAPPSIAAACGIDAFIHALEAYINT-AATPFTDAMGEKAMELIGANIRRFVAN    233
Csp-CpnD     LDAQLTIALPPAVTGATGVDALVHAIEALSTRLKRNPLSDLLAIKAVQMIYDHLPLAVKE    233
                 :    *  .  : .:**: *. *:       .  :.  .  . .           .

Zmob-AdhB    GKDM--PAREAMAYAQFLAGMAFNNASLGYVHAMAHQLGGY-----YNLPHGVCNAVLLP    286
Cnec-Hbd     GQDR--DARLNMMSASMQGAMAFQK-GLGCVHSLSHPLGGLKIDGRTGLHHGTLNAVVMP    289
Cklu-Hbd     GNDYRVEIIEDFVIGSNYAGIAFGNAGVGAVHALSYPIGGN-----YHVPHGEANYLFFT    276
Cvir-Hbd1    GPEARISLLEQFLTASNYAGIAFGNAGCAAVHALSYPLGAT-----YHVPHGEANYAIFT    275
Cvir-Hbd2    GQEARIPLLSDFLLAANYAGIAFGNAGCAAVHALSYPLGAV-----YHVPHGEANYAMFT    275
Cvir-Hbd3    GEEARFPILEQFLLASNYAGIAFGNAGCAAVHALSYPLGAV-----YHVPHGEANYAMFT    275
Cvir-Hvd     RKDE--AAACAMMLGSTFAGVAFAFARLGNIHAMSHPVSAF-----FHTPHGVANAVLMP    286
Csp-CpnD     GSNL--EVREQMLLASLFAGMAFSNASVGAVHAFAYPLGVR-----FHVPHGLSNSLMLP    286
               :        :  .   ..:**     . :*:::: :.           **  *  .:
```

Figure S1. Analysis of metal-binding motif in FeADHs. Iron-coordinating residues are marked in magenta, the putative catalytic base in green and the putative modulating Asn in violet.

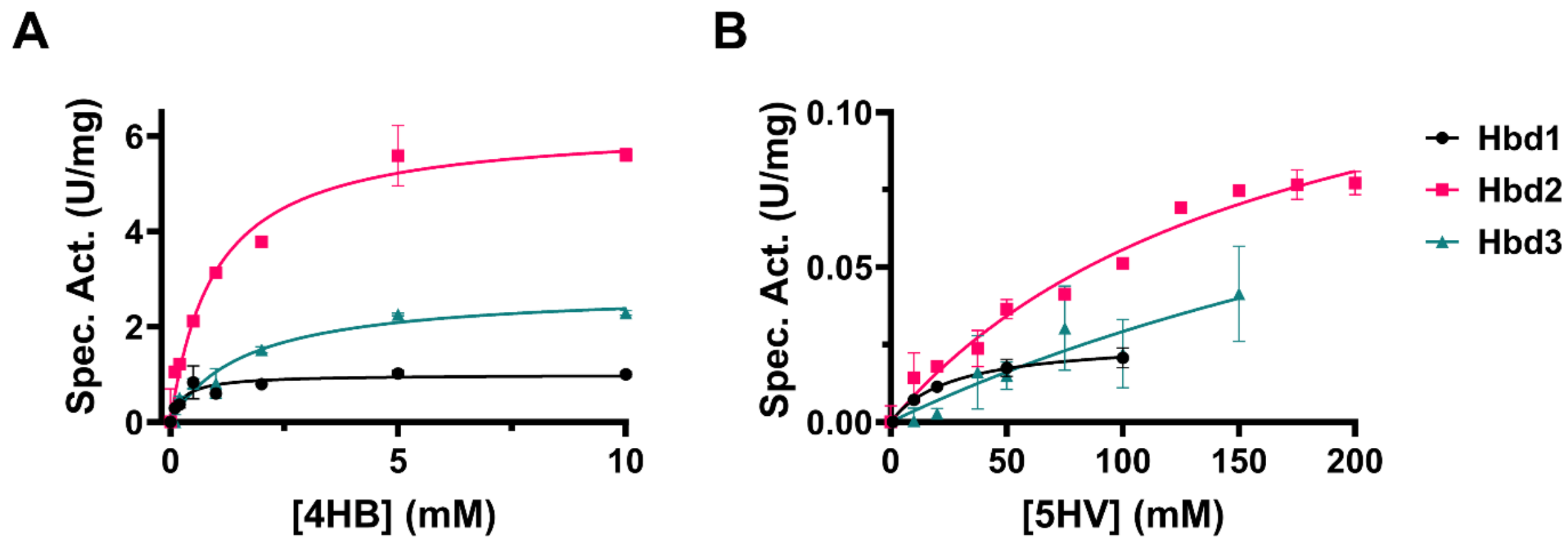

C

FeADH	$K_{m,\ 4HB}$ (mM)	$V_{max,\ 4HB}$ (U/mg)	$K_{m,\ 5HV}$ (mM)	$V_{max,\ 5HV}$ (U/mg)	Cat. Eff. $_{4HB}$ / Cat. Eff. $_{5HV}$
**Hbd1**	**0.2**	**1.0**	**30**	**0.03**	**5000**
**Hbd2**	**1.0**	**6.2**	**170**	**0.15**	**7000**
**Hbd3**	**1.6**	**2.8**	**> 150**	**0.10**	**> 2500**

Figure S2. Estimated kinetic parameters of Hbd1, Hbd2, and Hbd3 with 4-hydroxybutyrate and 5-hydroxyvalerate. Michaelis–Menten plots for the conversion of 4-hydroxybutyrate (**A**) and 5-hydroxyvalerate (**B**) by Hbd1, Hbd2, and Hbd3 with NAD^+ (1 mM, 100 mM Tris/HCl, pH 9). Apparent kinetic parameters were estimated (**C**). Enzymes were purified via Ni-NTA affinity chromatography, metal-depleted using EDTA and Mn^{2+}-reconstituted. Measurements were performed under aerobic conditions. Data are duplicates for one protein preparation with error bars indicating the variation between technical replicates.

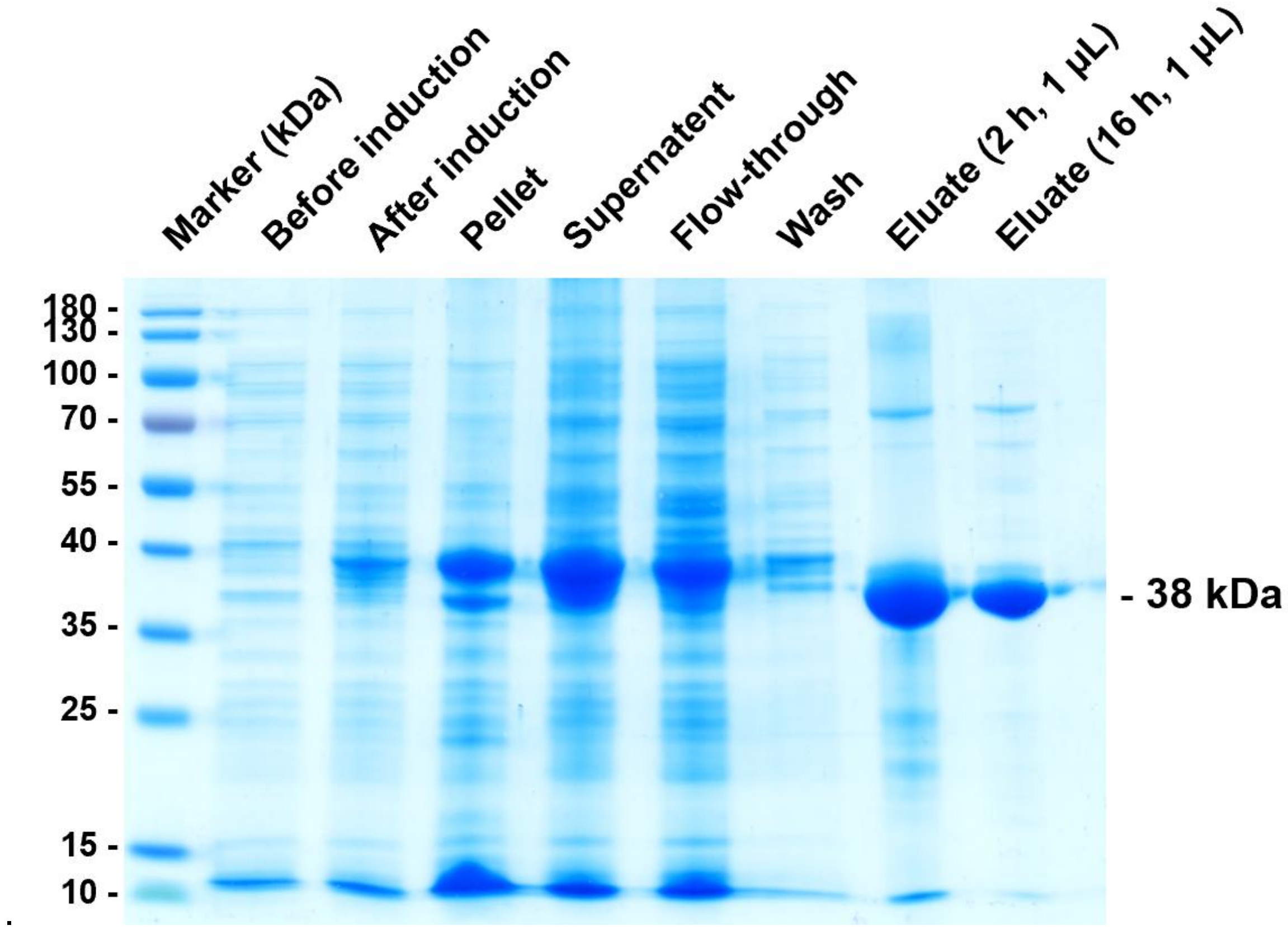

Figure S3. SDS-PAGE of Cvir-Hbd2 (wildtype) purification with Cytiva ProteinSelect.

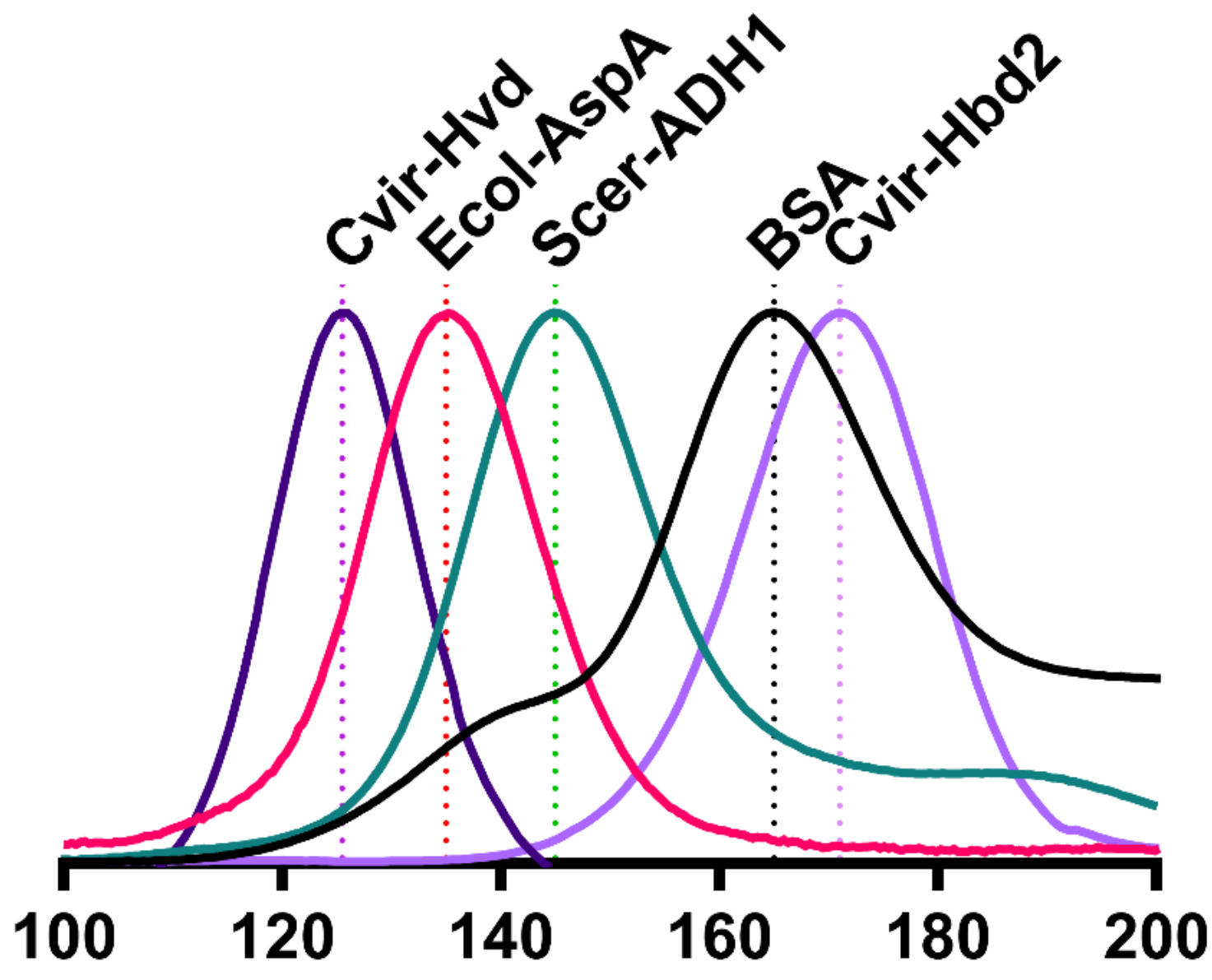

Figure S4. Gel filtration of Cvir-Hbd2 and Cvir-Hvd. For comparison of possible quaternary structures, separate runs were performed with aspartate ammonia lyase from *E. coli* (Ecol-AspA ; M ~ 210 kDa), alcohol dehydrogenase 1 from yeast (Scer-ADH1; M ~ 150 kDa) and bovine serum albumin (BSA; M ~ 66 kDa).

```
Zmob-AdhB       MASSTFYIPFVNEMGEGSLEKAIKDLNGSGFKNALIVSDAFMNKSGVVKQVADLLKAQGI  60
Cnec-Hbd        -MAFIYY-LTHIHLDFGAVSLLKSECERIGIRRPLLVTDKGVVAAGVAQRAIDA--MQGL  56
Cklu-Hbd        MKLLKLA--PD-VYKFDTAEEFMKYF-KV-GKGDFILTNEFLYKPFLEK-------FNDG  48
Cvir-Hbd1       MKQFVIK--PI-IYKFETCRDFVAQF-HL-SDDDLILTNEFIYAPFFGD-------LDLT  48
Cvir-Hbd2       MKEFSIK--PK-VIQMSNCKELASHF-QF-GPNDLILTNEYIFTPFFGN-------MSLP  48
Cvir-Hbd3       MTQFIIH--PR-IYQMASCEEFAAQV-QL-GAEDLILTNAYIYEPAFGS-------MHLP  48
Cvir-Hvd        MKDFNFNILQNCEFGMGALKKLPDILKKCGSDNVLLISDRGLEAIGVVGKVKAIIEEAGL  60
Csp-CpnD        MNPFHFETTPRIICHNGASTKLGEAARNLGIRHAFFVTDKGVHQAGLTSAAMDSLKAADI  60
                                                  :::::  :    .
```

Figure S5. Analysis of NAD(H)/NADP(H)-binding motif in FeADHs. Position D39 in Zmob-AdhB, associated with NAD(H)-specificity, is marked in magenta.

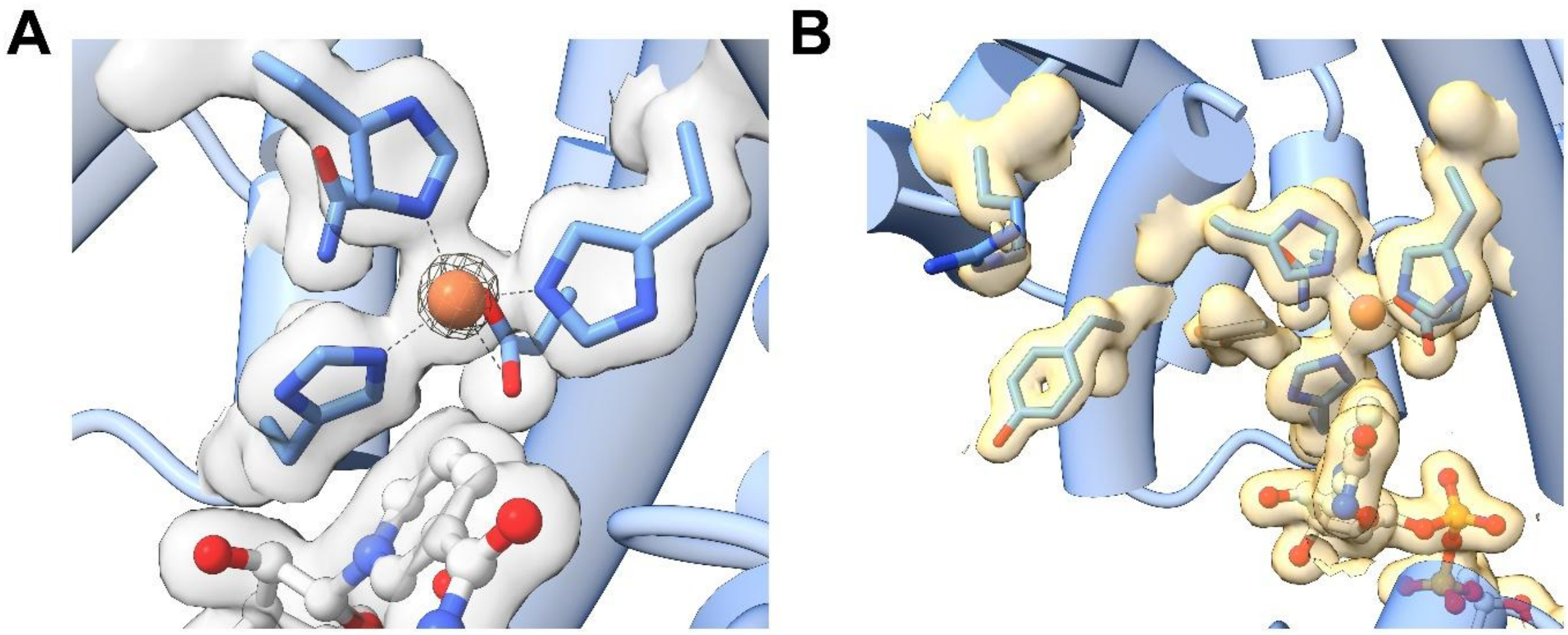

Figure S6. Electron density maps of Cvir-Hbd2. (**A**) The 2F_o-F_c electron density map is shown as a transparent silver surface (contour level 1.0 rmsd). The anomalous difference Fourier map calculated from data collected at the Fe K-edge (1.73 Å) is shown as an orange mesh (contour level 5.0 rmsd). (**B**) The 2F_o-F_c electron density map is shown as a transparent orange surface (contour level 1.0 rmsd). R348, located at the end of the tunnel, shows little electron density, indicating flexibility of its side chain.

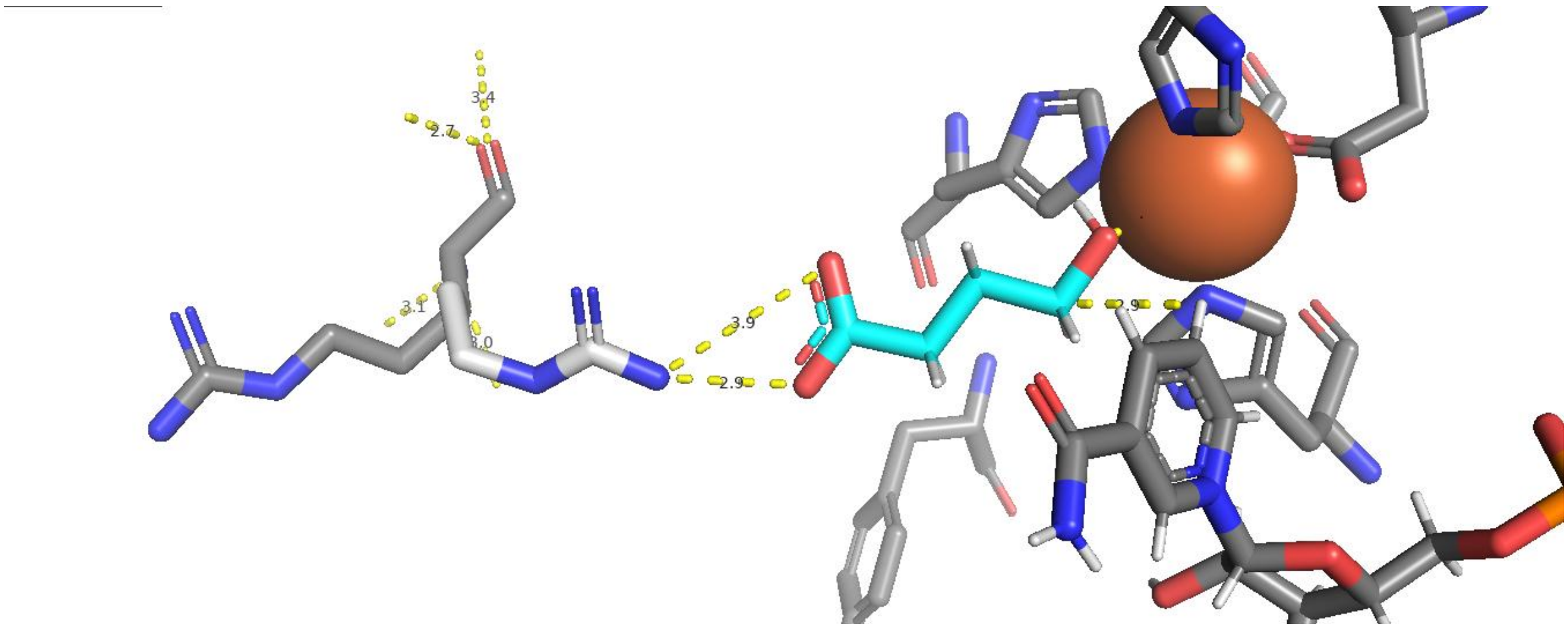

Figure S7. Modelling based on geometrical constraints of 4-hydroxybutyrate into the active site of Cvir-Hbd2. 4-Hydroxybutyrate (created in Avogadro) was positioned into the active site tunnel using PyMOL 3. A suitable rotamer of R348 was chosen to obtain geometrically consistent substrate binding.

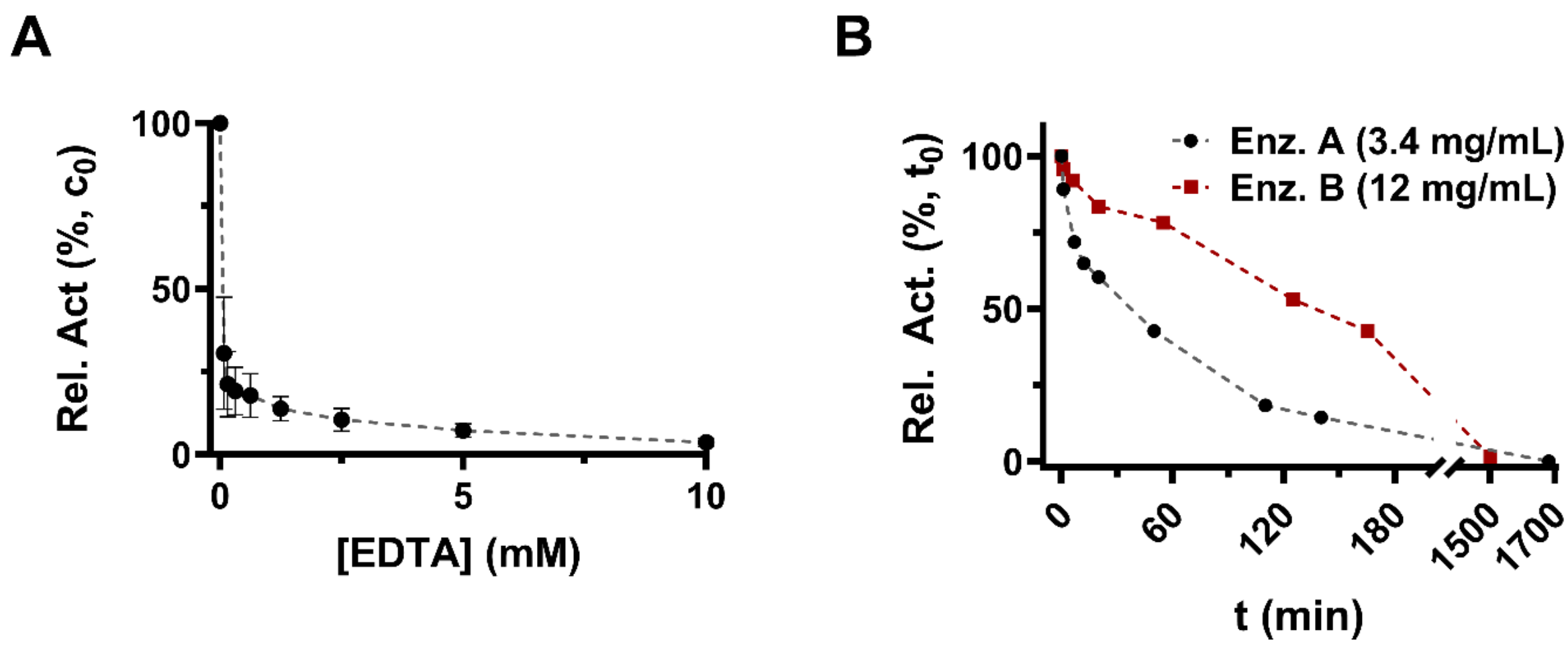

Figure S8. Effect of EDTA on Cvir-Hbd2 activity. **(A)** Cvir-Hbd2 (~1 mg/mL) was incubated anaerobically overnight with EDTA. **(B)** 10 mM EDTA was added to two different preparations of Cvir-Hbd2 at indicated protein concentrations. For both experiments, incubation and activity measurement were performed at 25 °C. Residual activity was determined by monitoring 4-hydroxybutyrate oxidation.

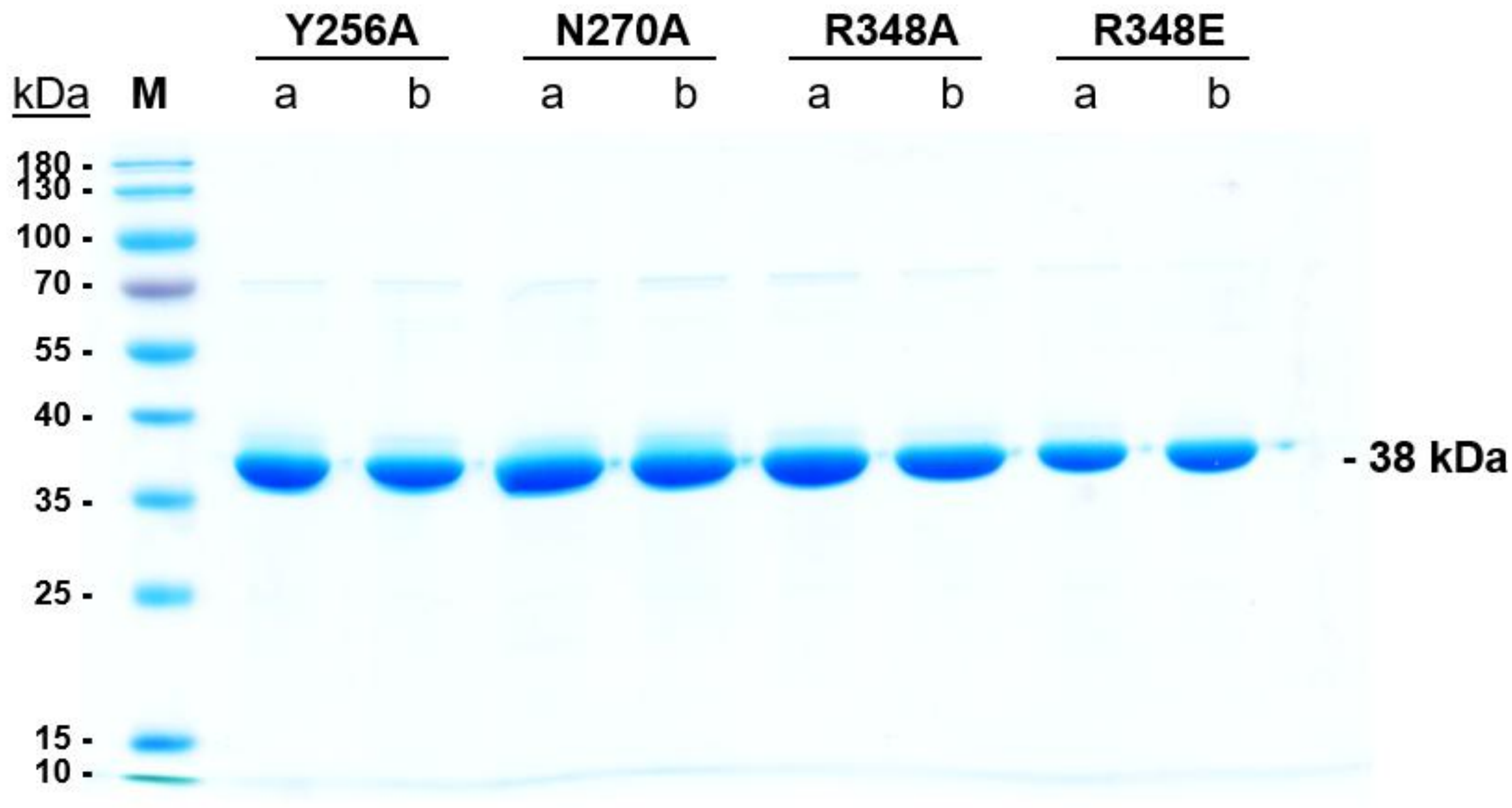

Figure S9. SDS-PAGE of purified Cvir-Hbd2 variants.

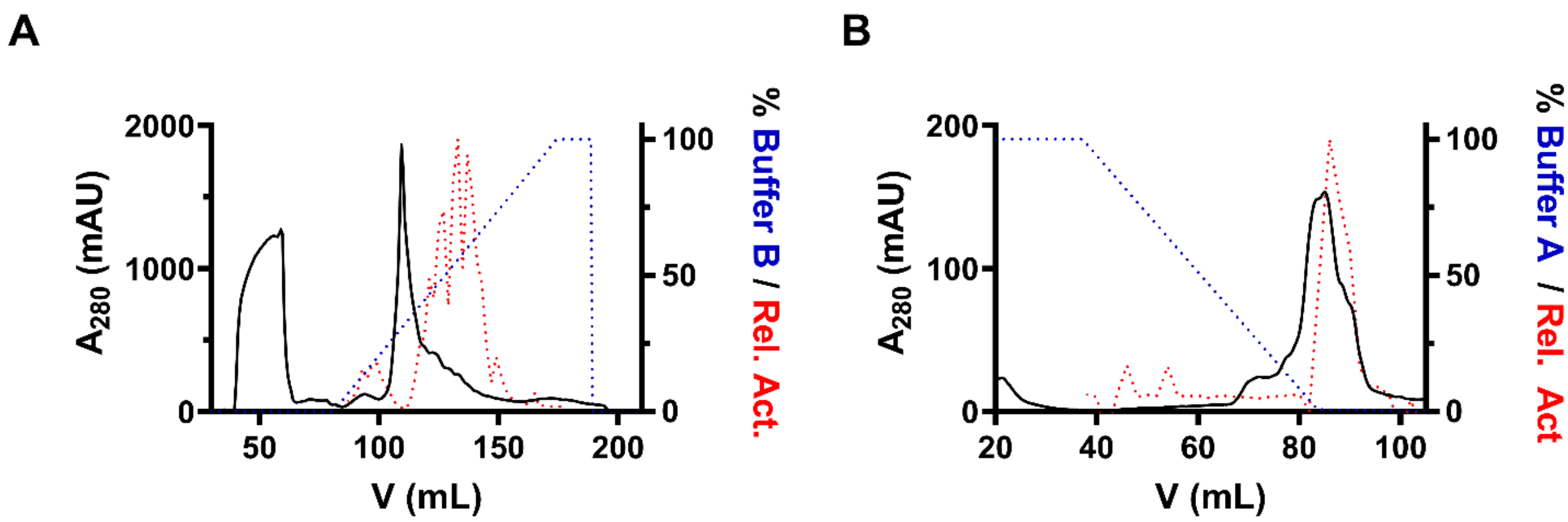

Figure S10. Purification of Cvir-Hvd from 5-aminovalerate grown *C. viride*. (**A)** Anion-exchange chromatography of *C. viride* supernatant on a HiScreen Q FF column. Bound proteins were eluted with a linear $(NH_4)_2SO_4$ gradient from 0 to 200 mM over 20 column volumes in 20 mM BisTris/HCl, pH 7.0. (**B)** Pooled peak-activity fractions from A (~ 110 mM $(NH_4)_2SO_4$ were applied to a HiScreen Phenyl FF (low sub) column after adjustment to 2 M $(NH_4)_2SO_4$. Bound proteins were eluted as described in A, with a 2000 – 0 mM gradient of ammonium sulfate. Fractions of 2 mL were collected and assayed for 5HV oxidation as described in the Materials and Methods section. Activities are shown relative to the fraction with maximum activity.

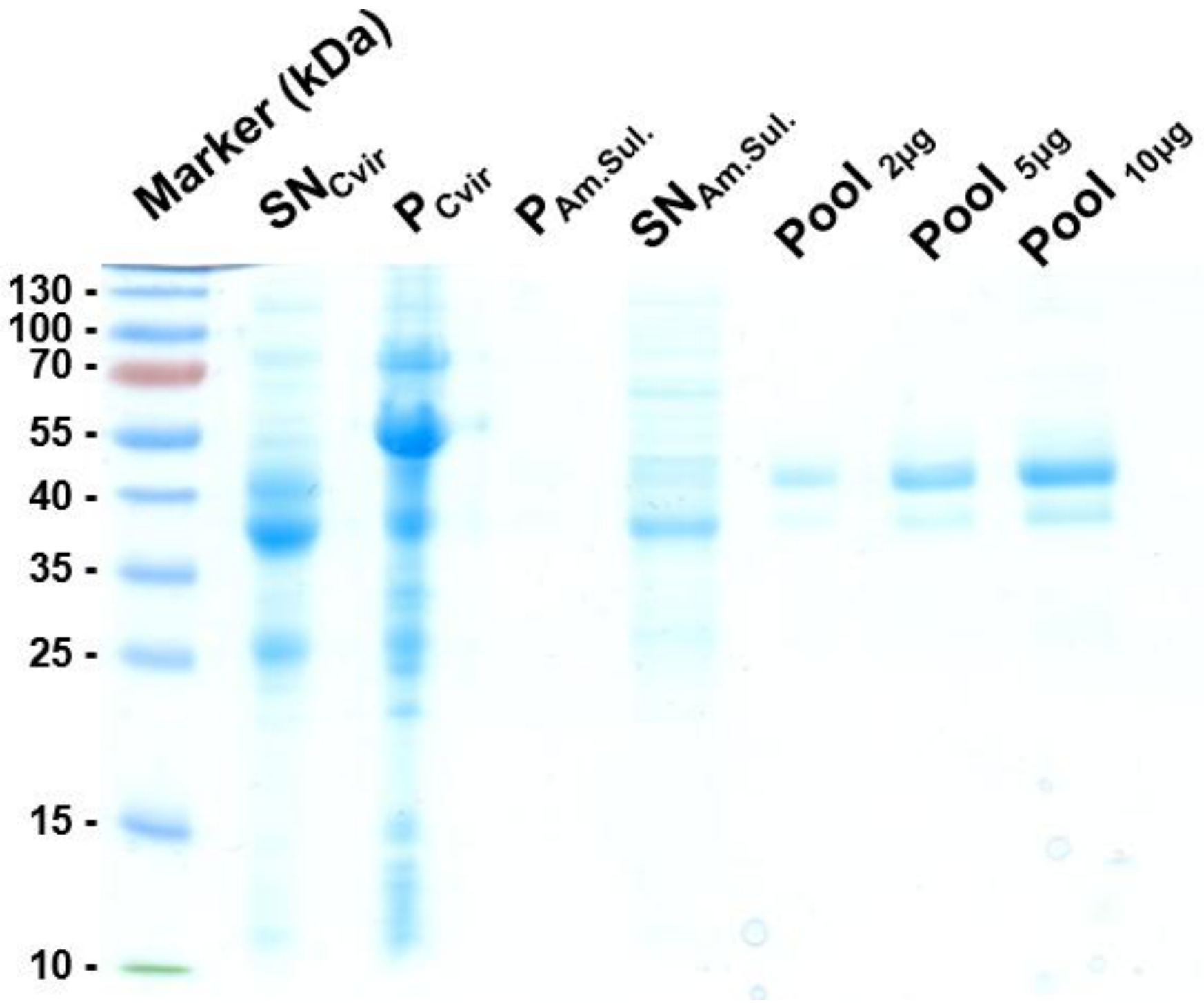

Figure S11. SDS-PAGE of the purification for 5-hydroxyvalerate dehydrogenase directly from *C. viride*. Shown are the soluble whole cell lysate, the pellet after centrifugation, the ammonium sulfate pellet after anion-exchange chromatography and salt adjustment, the soluble fraction applied to hydrophobic interaction chromatography, and pooled fractions containing Cvir-Hvd loaded at 2, 5, and 10 µg protein.

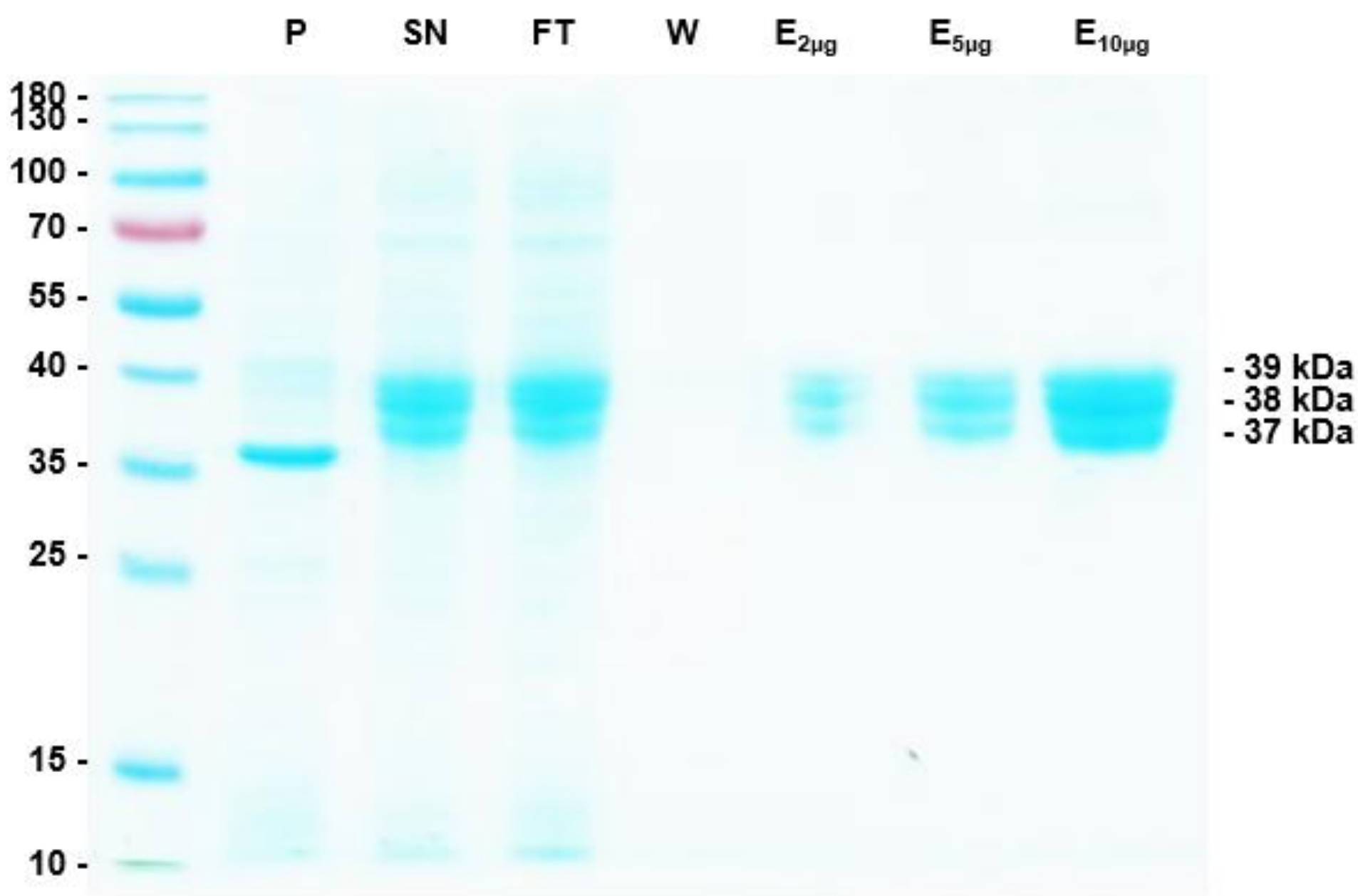

Figure S12. SDS-PAGE of Cvir-Hvd (WT) purification with StrepTactin XT using a TwinStrep tag. Note the multiple bands from partial proteolysis of the protein.

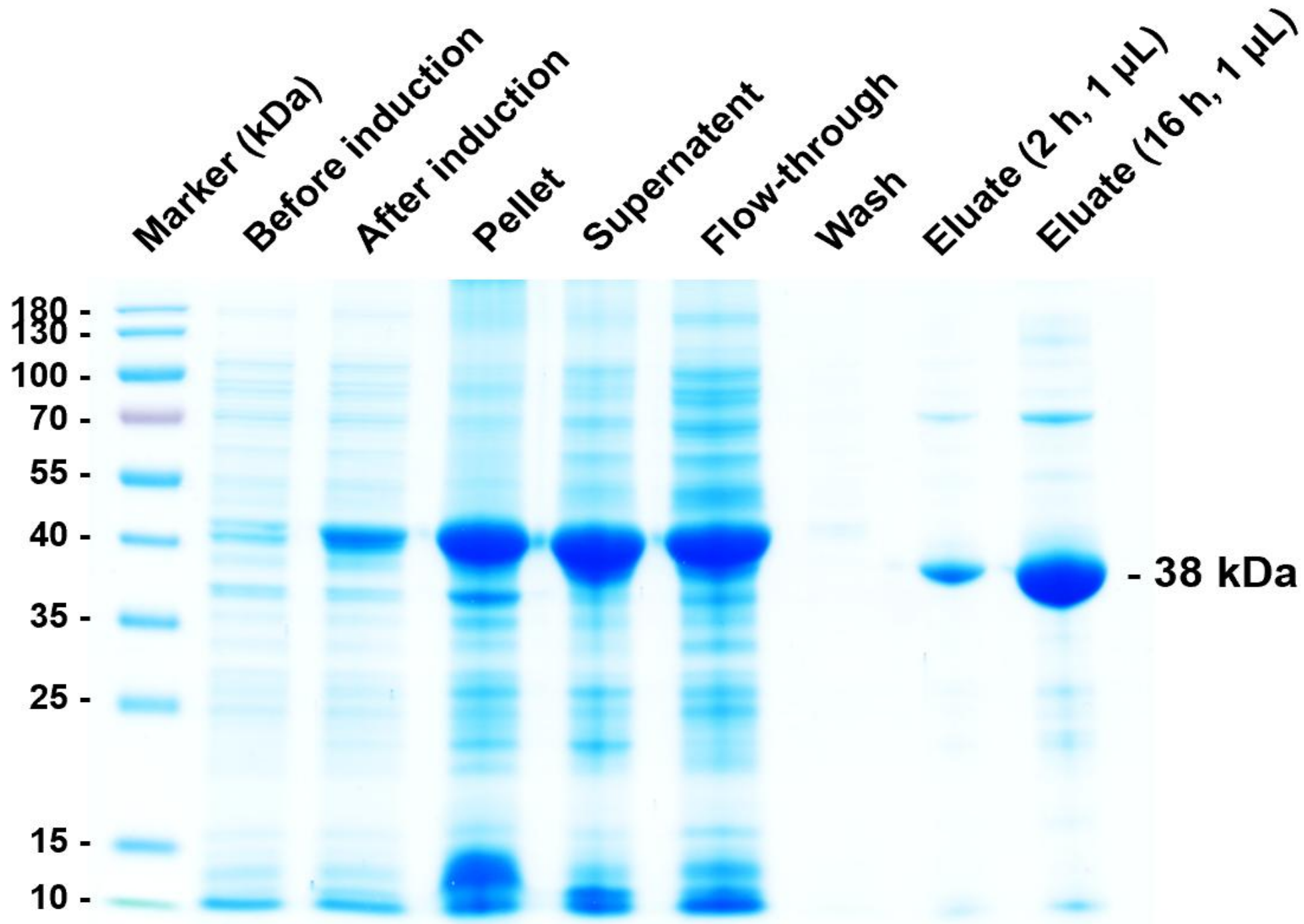

Figure S13. SDS-PAGE of Cvir-Hvd (WT) purification with Cytiva ProteinSelect.

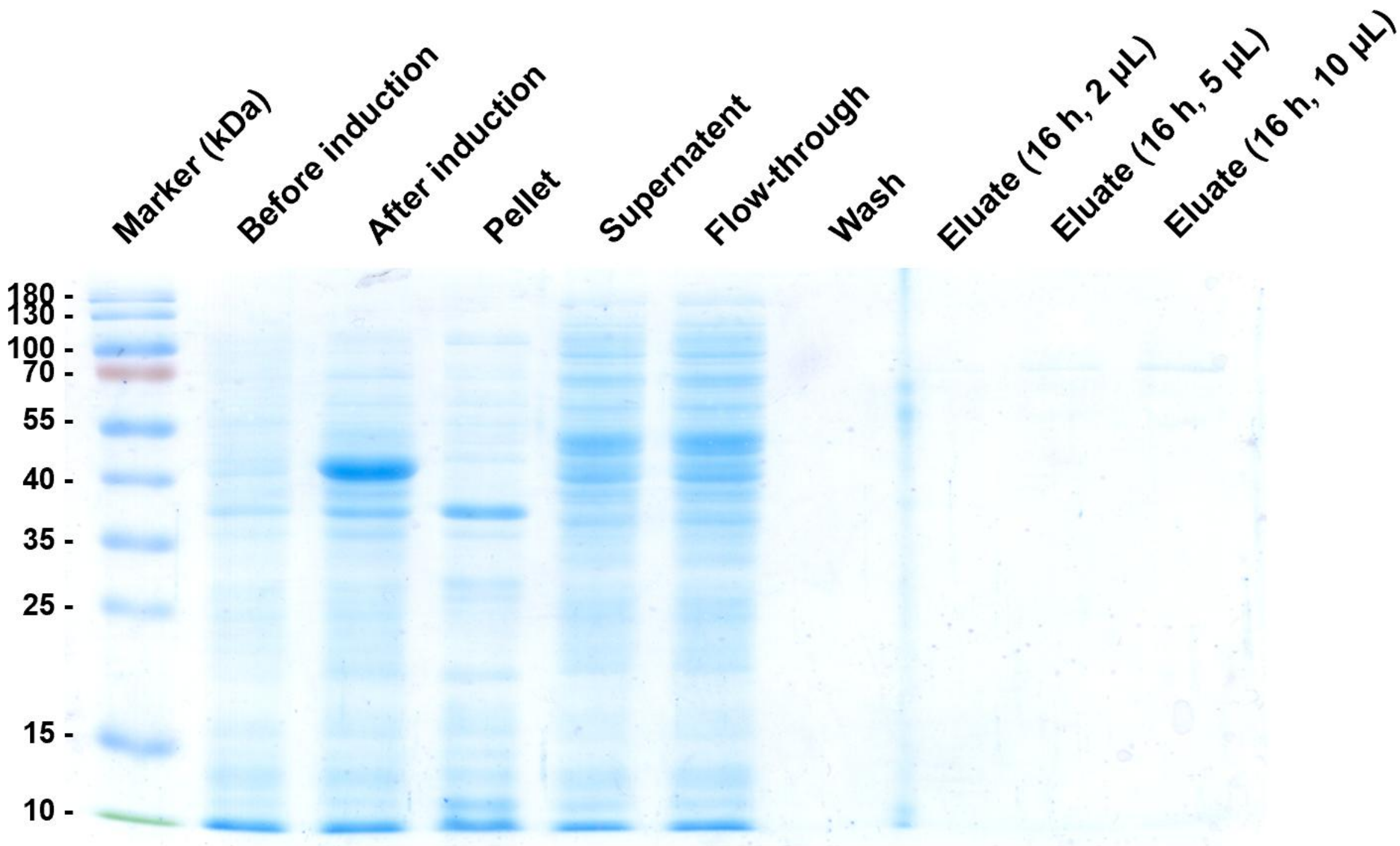

Figure S14. SDS-PAGE of Cvir-Hvd (D39G) purification with Cytiva ProteinSelect**.** No soluble protein was obtained, the pellet fraction indicated large amounts of inclusion bodies.

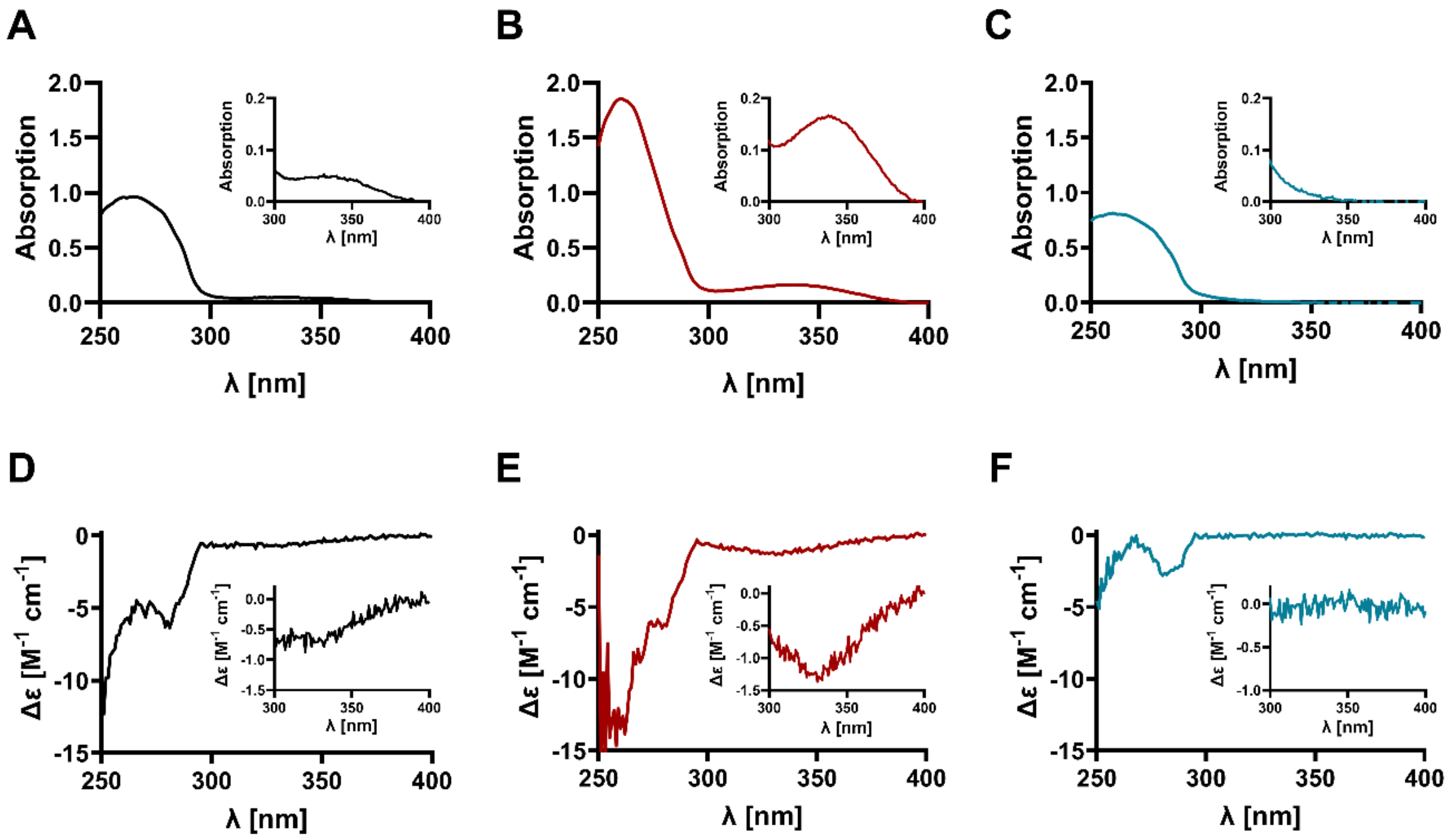

Figure S15. CD- and absorbance spectra of Cvir-Hvd. Absorbance spectra of purified Cvir-Hvd **(A)**, after addition of 20 μM NADH **(B)**, and after addition of 4.5 mM 5-oxovalerate **(C)**. The corresponding CD spectra are shown in **D–F**. After each addition, samples were incubated for 5 min at 25 °C. Insets in A-C highlight the absorbance spectrum of NADH, whereas the insets in D-F highlight the bound NADH giving rise to a negative Cotton effect. The spectra present the average of three scans.

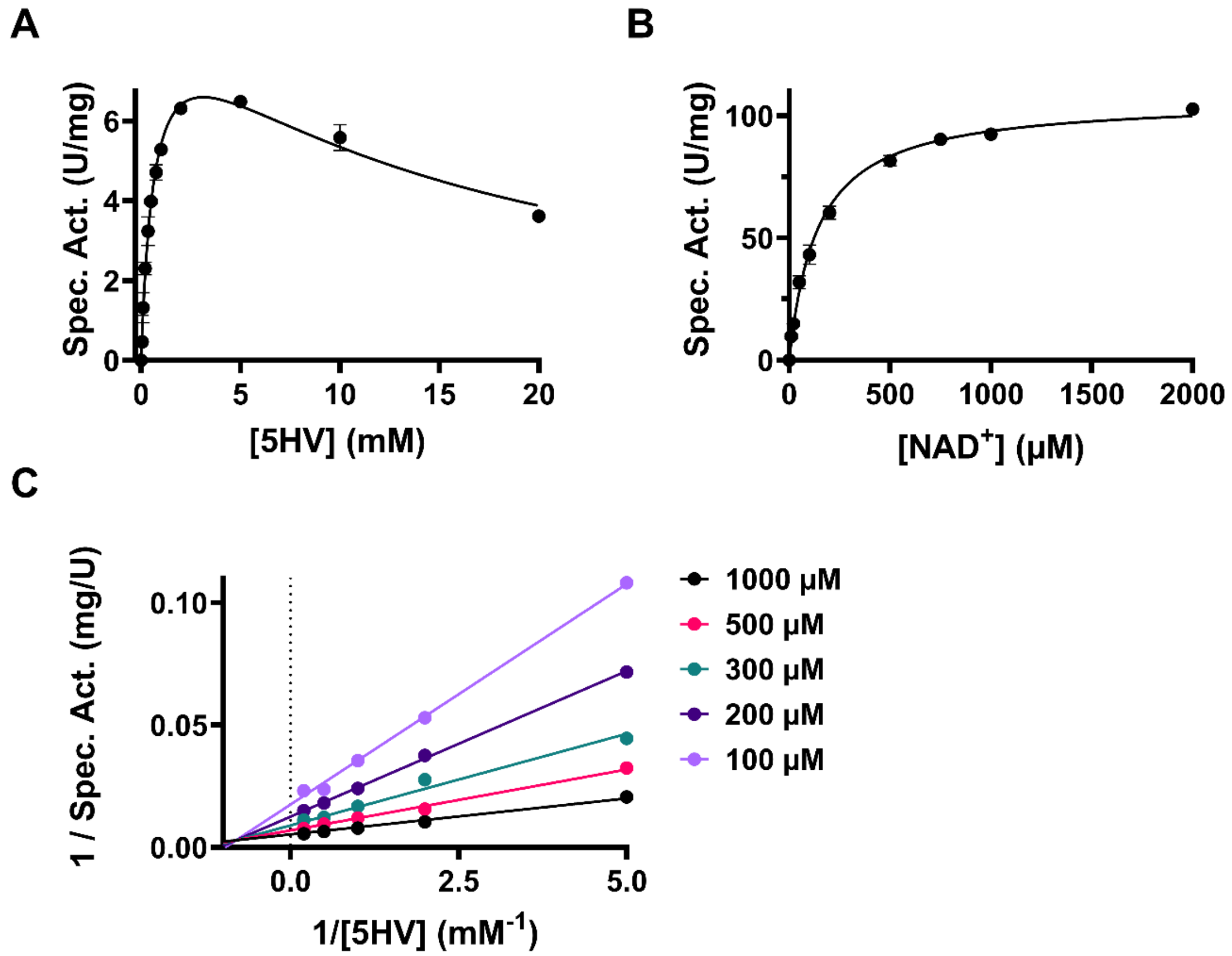

Figure S16. Extended kinetic data on Cvir-Hvd. **(A)** Michaelis–Menten analysis of 5HV oxidation by Mn^{2+}-reconstituted Cvir-Hvd under aerobic conditions (1 mM NAD^+, 100 mM Tris/HCl, pH 9.0). (**B**) Michaelis–Menten analysis of NAD^+ reduction by Fe^{2+}-reconstituted Cvir-Hvd (5 mM 5HV, 100 mM Tris/HCl pH 9.0), 100 mM Tris/HCl, pH 9.0). **(C)** Lineweaver–Burk plot of Fe^{2+}-reconstituted Cvir-Hvd with varying concentrations of both 5HV and NAD^+. The lines were modeled to intersect at a common point. Assays were performed in 100 mM Tris/HCl, pH 9.0, using 0.2–10 mM 5HV.

```
Zmob-AdhB     LGAKKEDVPLLADHALKDA-CALT-NPRQGDQKEVEELFLSAF----    383
Cnec-Hbd      MGVTEDMFDKVIAGALVDH-CHKT-NPKEASAADYRRMLEQSM----    382
Cklu-Hbd      YGMKEEEIETFADSVIEGQQRLLVNNYEPFSREDIVNTYKKLY----    371
Cvir-Hbd1     YGVTEANLMEFTISVRQNQQRLMSNNFVPLDDDAVYNIYRTLF*---    370
Cvir-Hbd2     YGMTETEIQSFTDSVRENQTRLMANNFVPLDDAAVYNIYRSLY*---    370
Cvir-Hbd3     YGMTEEDLQIFTNSVRENQQRLMANNFVPLSDEDVYMIYRGLYK*--    371
Cvir-Hvd      VGVTEDKIPAMAADAMKSG-NVLV-NPRSSTVKDIEMLYKKAMYVK*    384
Csp-CpnD      LGVERDDLAMLAKDVMGIQ-RLLDHNPCPITHEDAMKLYARAF----    384
               *  .  .  .   .          *
```

Figure S17. Analysis of the substrate binding motif. Position C362 (oxidized cysteine) in Zmob-AdhB and R348 in Cvir-Hbd2, both associated with substrate binding, are marked in magenta.

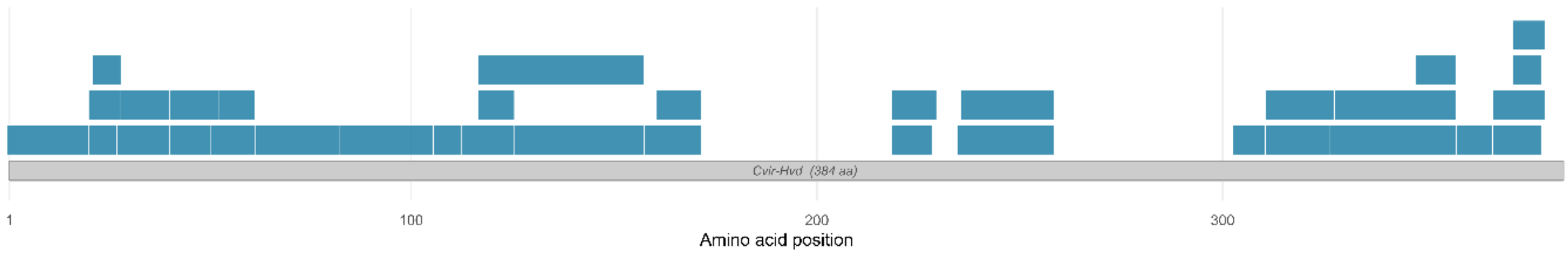

Figure S18. Peptide coverage map for Cvir-Hvd isolated from 5-aminovalerate grown *C. viride* cells. 34 unique peptides were identified, no razor peptides matched.

All synthetic genes were obtained from Twist Bioscience precloned into pET29b(+) using the NdeI and XhoI restriction sites. The start codon is contained within the NdeI recognition sequence (CAT**ATG**).

>Cvir-Hbd1-His6

GGTTCAAGTCACCACCACCACCACCATTCTTCTGAAAACTTGTACTTTCAGGGCCATATG
AAGCAATTCGTTATCAAGCCCATAATCTACAAGTTCGAGACATGTCGGGACTTTGTTGCA
CAATTTCATTTATCTGACGACGACTTAATACTTACTAATGAGTTCATCTACGCACCTTTCT
TTGGGGACTTGGACCTTACTTGTGACACTATCTTCCAAGAGAAGTACGGAGAGGGCGA
GCCAAGTGACGAGATGGTAGAGGCCATATACGCAGACATCAAGAAGGAGTATCAAAGA
ATCATAGCCATCGGAGGAGGAGCGGTTTTAGACATCGCGAAGGTCCTTGCGCTTAAGC
ACGTCTCACCTGTCCTTGCACTTTATGACGGTGAGAAGGAGATCATCAAGGAGAAGCAC
TTGATCTTGGTCCCTACTACTTGCGGGACGGGATCTGAGGTAACTAATATAAGTATTTTA
GCCTTAAACTCGCGCGGGACTAAGAAGGGTTTGGCGGCAGACGCTTTATTTGCCGACT
CTGCTGTCTTAATTCCCGAGCTTTTAGCTCGTTTGCCATTCCGTTTCTTCGCTACGTCTT
CCATCGATGCGCTCATCCATGCGATAGAGAGTACGCTCTCACCTAAGGCATCAGAGATC
ACGCAACTTTTCTCGTATAAGGCTATAGAGTTAATCTTAAATGGTTATTTGGAAATACGTG
ACCAAGGTCCCGAGGCTCGTATTTCGTTATTGGAGCAATTCTTGACCGCTTCGAACTAT
GCGGGAATCGCATTTGGCAATGCAGGCTGTGCTGCTGTCCACGCCCTTTCCTACCCATT
AGGGGCAACATATCATGTACCACACGGAGAGGCAAACTATGCAATTTTCACGGGTGTAA
TGAATCAATACATGTCCATTCGGAGTGACGGGGAGATAGCAAAATTGAATTCTTTCATCG
CTGAGATACTTGGCTGTGAGGTATCGCACGTATACGAGGAGTTAGAGACTTTATTAAAT
GTTCTCCTTCCTAAGAAGCCCTTAAATCAATACGGTGTAACAGAGGCTAATCTTATGGAG
TTCACTATCTCAGTTCGTCAAAATCAACAACGGCTTATGTCTAATAATTTCGTTCCCCTTG
ACGACGACGCGGTTTACAATATTTATCGTACTTTGTTTTGAGAGAATCTGTATTTCCAAG
GTCTCGAGGGTAGCGCGTGGTCTCATCCGCAGTTCGAGAAAGGCGGCGGGAGTGGTG
GTGGTTCTGGCGGGTCCGCCTGGTCACATCCACAATTTGAAAAGTGA

>Cvir-Hbd2-His6

GGATCCTCACACCACCACCACCACCATAGTAGTGAAAACTTGTACTTTCAGGGGCATAT
GAAGGAGTTCTCGATAAAGCCCAAGGTTATACAAATGTCGAACTGTAAAGAGCTTGCAA
GTCACTTCCAATTCGGTCCTAATGACCTTATCCTCACAAACGAGTATATATTCACACCCT
TCTTCGGCAACATGTCGCTTCCTTGTTGTGTCATCTTTCAAGAGAAGTACGGAAGTGGA
GAGCCATCTGACGCCATGGTTGACGCTATCTTACACGACCTTCCTTCTAACTTCCGGCG
TGTTATAGGGATCGGTGGTGGGGCGGTCTTGGACATCGCAAAGTTACTGGTCCTTCAAC
AAAACGGTTCCCTTCTTGACCTCTTCGACGGAAAGGTTCCCCCAGTCAAGGAGAAGGAG
TTGATACTCATACCCACTACGTGTGGGACCGGTTCGGAAGTTACTAATATCTCAATACTT
GCATTGCTTTCTCGTGGTACAAAGAAGGGTTTAGCCCACGAGGCTTTATTCGCGGACCA
AGCCGTATTAATACCTGAGCTCTTAGAGGGACTCCCATTTCGGTTCTTCGCTACTTCTTC
TATCGACGCCCTCGTTCACGCTGTCGAGAGTAGTCTGAGTCCAAAGGCTAGTGACCTTT
CAAAGTTATTCGGTTACCGTGCCATAGACATGATCTTACGAGGATTCTTGACGATACGG
GATCAAGGACAAGAGGCCCGTATCCCCTTATTATCGGACTTCCTTCTTGCCGCAAATTAT
GCAGGTATCGCGTTCGGTAATGCTGGCTGTGCAGCAGTCCACGCATTAAGCTATCCATT
AGGTGCGGTATACCACGTACCACACGGAGAGGCCAACTACGCAATGTTCACTGGCGTT
TTGAAGAAGTACCTCTCCATTCGGACAGACGGGGAGATAGCACACCTTAATGCCTTCAT
GGCCGACATCCTTGGCTGCCACCCTGACACAGTTTATGACAACTTAGAGGACCTTCTTA
ATGTCTTAATCCCTAAGCGTCCATTGAGAGAGTACGGTATGACCGAGACAGAGATCCAA
AGTTTTACAGACTCCGTTCGAGAGAACCAAACTCGTCTCATGGCTAATAACTTCGTTCCC
TTGGACGACGCGGCCGTATATAATATCTACAGATCTCTCTATTGAGAGAATCTGTATTTC
CAAGGTCTCGAGGGTAGCGCGTGGTCTCATCCGCAGTTCGAGAAAGGCGGCGGGAGT
GGTGGTGGTTCTGGCGGGTCCGCCTGGTCACATCCACAATTTGAAAAGTGA

>Cvir-Hbd3-His6

GGATCTTCACACCACCACCACCACCATTCATCCGAGAATCTTTATTTCCAAGGTCATATG
ACACAATTCATAATCCATCCACGGATCTACCAAATGGCTTCTTGTGAAGAGTTTGCAGCG
CAAGTTCAACTCGGTGCTGAGGACCTTATATTAACGAACGCATATATATACGAGCCAGC
CTTCGGCAGTATGCACTTACCATGCCACGTTATATACCTCGAAAATTACGGTAGTGGGG
AGCCCTCAGACGAGATGGTTGAGGCCATATACCAAGACTTGCCAGCATCCTTCCGTCGT
GTAGTTGCCATAGGTGGTGGCGGGGTACTTGACGTTGCTAAGATCTTATCGCTCAAGCA
TATATCCCCTGTACTCGACTTATTCGATGGTAAGACAGAGGTCATCCGTGACAAGGAGC
TTATCTTGGTACCCACCACGTGTGGCACGGGTTCAGAGGTCACTAACATATCTATTCTTG
CATTGAATTCTAGAGGAACAAAGAAGGGTCTTGCCACGGAAGCTTTATTCGCTGACTCT
GCTGTACTCATACCTGCATTGCTTATGGGGCTTCCCTTCCGTTTCTTCGCGACTTCTGCA
ATAGACGCCTTAGTTCACGCCGTTGAGAGTTCACTTAGTCCAAAGGCCTCTGCAGCAAG
CAAGTTGTTCTCTTATCAAGCGATTGATATGATCTTAAGAGGTTTCTTAGAGATACGTAAT
CAAGGTGAAGAGGCACGCTTTCCTATCCTCGAACAATTTCTGCTTGCAAGTAATTACGCT
GGGATCGCTTTTGGAAACGCAGGGTGTGCTGCAGTTCACGCACTCAGTTACCCCCTCG
GTGCTGTATACCACGTTCCTCACGGGGAAGCTAACTATGCTATGTTCACCGGGGTCATG
AATAAGTACTTGTCAATACGCTCCGACGGCGAGATCTCTCACTTGAATGACTTCATAGCC
AATATACTTCAATGCGACGTTTCCTGTGTTTACGAGGAGTTAGAGAATCTCTTAAATGTC
TTGATCCCAAAGAAGCCTTTGTTCGAGTACGGGATGACGGAAGAGGATTTACAAATCTT
CACTAACTCTGTTCGTGAGAACCAACAACGCTTAATGGCAAACAATTTCGTGCCTTTGTC
CGACGAGGACGTTTATATGATATATCGTGGTCTTTATAAGTGAGAGAATCTGTATTTCCA
AGGTCTCGAGGGTAGCGCGTGGTCTCATCCGCAGTTCGAGAAAGGCGGCGGGAGTGG
TGGTGGTTCTGGCGGGTCCGCCTGGTCACATCCACAATTTGAAAAGTGA

>Cvir-Hbd2-CytivaSelect

GTAAAGATAGTCAGTCGGAAGAGCCTAGGCGTGCAGAACGTTTATGATATTGGCGTGGA
GAAAGATCATAACTTTCTGCTCGCGAACGGTCTTATTGCCAGCAACAAATTCGAAAAGG
AGTTCTCGATAAAGCCCAAGGTTATACAAATGTCGAACTGTAAAGAGCTTGCAAGTCACT
TCCAATTCGGTCCTAATGACCTTATCCTCACAAACGAGTATATATTCACACCCTTCTTCG
GCAACATGTCGCTTCCTTGTTGTGTCATCTTTCAAGAGAAGTACGGAAGTGGAGAGCCA
TCTGACGCCATGGTTGACGCTATCTTACACGACCTTCCTTCTAACTTCCGGCGTGTTATA
GGGATCGGTGGTGGGGCGGTCTTGGACATCGCAAAGTTACTGGTCCTTCAACAAAACG
GTTCCCTTCTTGACCTCTTCGACGGAAAGGTTCCCCCAGTCAAGGAGAAGGAGTTGATA
CTCATACCCACTACGTGTGGGACCGGTTCGGAAGTTACTAATATCTCAATACTTGCATTG
CTTTCTCGTGGTACAAAGAAGGGTTTAGCCCACGAGGCTTTATTCGCGGACCAAGCCGT
ATTAATACCTGAGCTCTTAGAGGGACTCCCATTTCGGTTCTTCGCTACTTCTTCTATCGA
CGCCCTCGTTCACGCTGTCGAGAGTAGTCTGAGTCCAAAGGCTAGTGACCTTTCAAAGT
TATTCGGTTACCGTGCCATAGACATGATCTTACGAGGATTCTTGACGATACGGGATCAA
GGACAAGAGGCCCGTATCCCCTTATTATCGGACTTCCTTCTTGCCGCAAATTATGCAGG
TATCGCGTTCGGTAATGCTGGCTGTGCAGCAGTCCACGCATTAAGCTATCCATTAGGTG
CGGTATACCACGTACCACACGGAGAGGCCAACTACGCAATGTTCACTGGCGTTTTGAAG
AAGTACCTCTCCATTCGGACAGACGGGGAGATAGCACACCTTAATGCCTTCATGGCCGA
CATCCTTGGCTGCCACCCTGACACAGTTTATGACAACTTAGAGGACCTTCTTAATGTCTT
AATCCCTAAGCGTCCATTGAGAGAGTACGGTATGACCGAGACAGAGATCCAAAGTTTTA
CAGACTCCGTTCGAGAGAACCAAACTCGTCTCATGGCTAATAACTTCGTTCCCTTGGAC
GACGCGGCCGTATATAATATCTACAGATCTCTCTATTGATGAGAGAATCTGTATTTCCAA
GGTCTCGAGGGTAGCGCGTGGTCTCATCCGCAGTTCGAGAAAGGCGGCGGGAGTGGT
GGTGGTTCTGGCGGGTCCGCCTGGTCACATCCACAATTTGAAAAGTGA

>Cvir-Hvd-TwinStrep

GCTAGCTGGTCTCATCCGCAGTTCGAGAAAGGCGGCGGGAGTGGTGGTGGTTCTGGC
GGGTCCGCCTGGTCACATCCACAATTTGAAAAGGAGAATCTGTATTTCCAAGGGAGTGG
CAGCATGAAGGACTTCAATTTCAACATCTTACAGAACTGCGAATTTGGCATGGGCGCAT
TGAAAAAACTGCCGGATATTTTAAAAAAATGCGGCTCCGATAACGTCCTGCTGATTAGC
GATCGCGGCTTAGAAGCGATTGGCGTGGTGGGCAAAGTGAAAGCGATTATTGAGGAAG
CGGGCCTGAAATATAACGAGTACCTGGATGTGGTGCCGAACCCGACCACCGACGTGGT
GGAAGCGTGCGGGAAACTGTATGCCGAATGCGGCGCGACCAGCATGCTGGCACTGGG
TGGTGGCAGTCCGATGGATGTGGCGAAAGTGGTGGGCGTGGTGGCGCGCTACGGCGG
CAAAGTGACCGATTACGAAGGCGTGGGCAAAGTGCCGGGCCCGATTGATACCCTGATT
GCGATTCCGACCACCGCGGGTACCGGCAGCGAAGTCACCGTGGCGGCTGTGATTACC
GATGAAGCGCGGAACTACAAACTGAGCGTTCTGTCCCCGGAAATTAGCCCGAAATACG
CGGTCCTGGATCCGGAATTAATTATGACCGCGCCACCGAGCATTGCCGCGGCGTGCGG
CATCGATGCGTTTATTCATGCGTTAGAAGCGTACATTAACACCGCGGCGACGCCGTTTA
CCGATGCCATGGGCGAAAAAGCGATGGAACTGATTGGTGCGAATATTCGCCGCTTTGTT
GCGAACCGTAAAGATGAAGCGGCGGCGTGCGCGATGATGCTGGGCAGCACCTTTGCA
GGTGTGGCGTTTGCCTTTGCCCGGCTGGGCAACATTCATGCGATGAGTCATCCAGTGT
CCGCTTTTTTTCATACCCCGCATGGTGTGGCGAACGCCGTCCTGATGCCGACCATTCTG
GAATACAATGCGCTGGCCGACGAAGGCCGTTATGCGAAAGTGTACAAATATGTTACCGG
CAAAGATGCCGGCGCGGATTTTGTGCCGATGATGCTGGTGGATGAAATCCGTAAACTG
AACGCGGATCTGGGCATTCCGGCGTGCCTGGCGGACGTTGGCGTGACCGAAGATAAAA
TTCCGGCGATGGCGGCGGATGCCATGAAAAGTGGCAACGTTCTGGTGAACCCGCGGA
GCAGCACCGTCAAAGATATTGAAATGCTGTACAAAAAAGCGATGTACGTAAAATAA

>Cvir-Hvd-CytivaSelect

GTAAAGATAGTCAGTCGGAAGAGCCTAGGCGTGCAGAACGTTTATGATATTGGCGTGGA
GAAAGATCATAACTTTCTGCTCGCGAACGGTCTTATTGCCAGCAACAAATTCGAAAAGG
ATTTTAACTTCAATATACTTCAAAATTGTGAGTTCGGGATGGGTGCGCTGAAGAAGCTCC
CTGACATCCTGAAGAAGTGTGGTAGTGACAATGTGCTTCTTATAAGTGACCGAGGTCTT
GAGGCAATCGGGGTAGTCGGGAAGGTAAAGGCCATAATCGAAGAGGCTGGGCTTAAGT
ACAATGAATATTTAGACGTTGTTCCCAATCCTACAACAGATGTAGTTGAGGCCTGTGGTA
AGCTTTACGCTGAGTGTGGGGCAACGTCAATGTTGGCCTTAGGCGGCGGTTCGCCTAT
GGACGTTGCAAAGGTCGTTGGAGTTGTTGCTCGGTATGGTGGTAAGGTAACAGACTATG
AAGGGGTAGGGAAGGTTCCTGGTCCTATCGACACTTTAATCGCAATACCAACGACAGCT
GGCACAGGGAGTGAGGTTACTGTAGCCGCCGTAATAACTGACGAGGCCAGAAATTATA
AGTTAAGTGTCTTGAGCCCTGAGATATCTCCCAAGTATGCTGTGTTGGACCCAGAGTTG
ATCATGACGGCACCGCCTTCAATCGCAGCCGCTTGTGGTATTGACGCCTTCATACACGC
TCTCGAAGCATATATCAATACTGCCGCCACACCTTTCACTGACGCAATGGGTGAAAAGG
CCATGGAGTTAATAGGGGCCAACATAAGACGTTTCGTGGCAAATCGAAAGGACGAGGC
AGCTGCCTGTGCTATGATGCTTGGATCCACGTTCGCCGGGGTCGCATTCGCATTCGCA
AGATTAGGTAATATCCACGCCATGTCCCACCCTGTATCAGCGTTCTTTCACACACCCCA
CGGCGTCGCCAATGCTGTATTGATGCCAACGATCCTTGAGTATAACGCCTTAGCAGATG
AGGGACGGTACGCCAAGGTCTATAAGTACGTAACAGGGAAGGACGCAGGAGCCGACTT
CGTTCCAATGATGTTAGTAGACGAGATACGCAAGTTAAATGCTGACTTAGGGATCCCTG
CCTGTTTAGCCGATGTGGGTGTTACTGAGGACAAGATCCCTGCCATGGCTGCCGACGC
TATGAAGTCCGGTAATGTACTCGTTAATCCTCGTTCATCTACAGTGAAGGACATCGAGAT
GTTGTATAAGAAGGCAATGTATGTTAAGTGATAG
